# Sustainable EVA-based hybrid bio-composites derived from agricultural waste for efficient heavy metal removal

**DOI:** 10.1038/s41598-026-62480-9

**Published:** 2026-07-20

**Authors:** Kholod H. Kamal, Mahmoud E. Abd El-Aziz, Ahmed A. Haroun

**Affiliations:** 1 Water Pollution Research Department, National Research Centre, 33 El Bohouth Str, P.O. 12622, Dokki, Giza Egypt; 2 Polymers and Pigments Department, National Research Centre, 33 El Bohouth Str, P.O. 12622, Dokki, Giza Egypt; 3 Chemical Industries Research Institute, National Research Centre, 33 El Bohouth Str, P.O. 12622, Dokki, Giza Egypt

**Keywords:** Thermoplastics, EVA copolymer, Hybrid composites, Adsorption isotherm, Toxic metal ions, Chemistry, Engineering, Environmental sciences, Materials science

## Abstract

This study investigates ethylene-vinyl acetate (EVA)-based hybrid bio-composites for the effective removal of Cu^2+^ and Pb^2+^ ions from aqueous media. Two composites were fabricated via melt blending: C1 (EVA/biochar) and C2 (EVA/sugar beet pulp), utilizing agricultural wastes as functional fillers. Comprehensive characterization using SEM–EDX, FTIR, TGA, and DSC confirmed the successful incorporation of fillers and strong interfacial compatibility within the EVA matrix. Adsorption performance was evaluated by examining the effects of contact time, adsorbent dosage, and initial metal concentration. Both composites demonstrated rapid uptake, achieving equilibrium within 30 min for Cu^2+^ and 60 min for Pb^2+^. The maximum adsorption capacities reached 250 and 588 mg/g for Cu^2+^, and 97 and 82 mg/g for Pb^2+^ using C1 and C2, respectively. Kinetic and isotherm equilibrium behaviors were comprehensively evaluated using both linear and non-linear regression frameworks to thoroughly investigate the underlying adsorption mechanisms. The synergistic integration of the EVA matrix with bio-based fillers significantly enhanced surface functionality, structural integrity, and adsorption efficiency. Overall, these findings highlight the potential of EVA-based hybrid bio-composites derived from agricultural waste as efficient, sustainable, and cost-effective materials for heavy metal removal in water treatment applications.

**Supplementary Information:**

The online version contains supplementary material available at 10.1038/s41598-026-62480-9.

## Introduction

The persistent discharge of anthropogenically derived heavy metals and organic pollutants into aquatic ecosystems represents a critical global environmental challenge^1,2^. Unlike organic pollutants, metallic species such as Cu^2+^, Pb^2+^, Cd^2+^, and Hg^2+^ are non-biodegradable and prone to biomagnification within the food chain, posing severe neurotoxic and carcinogenic risks even at trace concentrations^3–6^. The proliferation of these contaminants is largely driven by industrial effluents from electroplating, mining, battery manufacturing, and metallurgical processing, as well as agrochemical runoff. Consequently, developing high-performance, cost-effective, and ecologically sustainable technologies for the selective removal of these ions remains a paramount research priority. Consequently, research efforts have increasingly focused on developing efficient, cost-effective, and environmentally friendly systems for heavy metal removal^7–10^.

To address heavy metal adsorption, several physicochemical methodologies have been deployed, including chemical precipitation^11^, membrane filtration^12^, ion exchange^5^, electrochemical treatment^13^, and adsorption^14^. Because of its ease of use, high efficiency, adaptability, and potential for regeneration, adsorption is generally acknowledged as one of these methods’ most alluring features. However, the industrial scalability of traditional sorbents such as activated carbon, mineral clays, and raw biomass is often hindered by poor mechanical durability, low selectivity, and technical difficulties regarding post-treatment recovery^15–18^. Recent advancements have shifted focus toward hybrid organic-inorganic composites to mitigate these drawbacks. By integrating inorganic or bio-based fillers into polymeric matrices, these systems synergistically combine the structural processability of the polymer with the high surface area and chemical functionality of the filler, yielding enhanced stability and tunable surface chemistry^19–23^.

Ethylene/vinyl acetate (EVA) is a versatile thermoplastic copolymer characterized by its chemical resilience, flexibility, and ease of thermal processing. The presence of polar vinyl acetate (VA) segments within the hydrophobic polyethylene backbone facilitates compatibility with hydrophilic reinforcements and offers potential coordination sites for metallic cations. Nevertheless, pristine EVA exhibits negligible inherent adsorption capacity and limited wettability, necessitating functional modification for water purification tasks^24,25^. Enhancing the adsorptive profile of EVA can be achieved through the incorporation of functional additives such as graphene oxide^26^, biochar^27^, layered silicates, natural zeolites^24^, or lignocellulosic agricultural wastes^9^. Biochar provides a porous, carbonaceous framework enriched with oxygenated functional groups conducive to ion exchange. Similarly, lignocellulosic waste, such as sugar beet pulp (SBP), is replete with hydroxyl, carboxyl, and phenolic moieties capable of forming stable complexes with divalent metals. Utilizing these agricultural by-products not only augments adsorption performance but also aligns with the principles of circular economy and waste valorization^28,29^.

The novelty and significance of this study lie in the fabrication of sustainable EVA-based hybrid bio-composites via a melt-blending technique reinforced with agricultural wastes (biochar and sugar beet pulp) as efficient adsorbents for Cu^2+^ and Pb^2+^ removal from wastewater. Its innovation surpasses traditional powdered biochar by embedding these fillers in a thermoplastic EVA matrix, yielding mechanically robust, flexible, and easily recoverable bio-composites. The synergy of EVA’s polar groups and biomass-derived oxygen functionalities enhances interfacial compatibility and adsorption performance. A multi-analytical approach, including FTIR, SEM–EDX, TGA, and DSC, was employed to characterize the structural, morphological, and thermal properties of the resulting bio-composites. Furthermore, the adsorptive efficacy toward Cu^2+^ and Pb^2+^ was rigorously evaluated through thermal and kinetic modeling studies. This work aims to demonstrate a synergistic strategy for developing mechanically robust and environmentally benign adsorbents, providing a viable pathway for the advanced remediation of heavy metal-laden wastewater.

## Materials and methods

### Materials

Residues of sugar beet after juicing were obtained from Egyptian Sugar and Integrated Industries Company (ESIIC) and it has been used as it is. The nitric acid (HNO_3_), copper sulfate, and lead acetate were obtained from Al-Gomhoria Co., Cairo, Egypt. Poly(ethylene-co-vinyl acetate) (EVA) was purchased from Sigma-Aldrich.

### Preparation of biochar

Casuarina (*Casuarina equisetifolia*) tree wastes were collected from a farm located in El-Menofia Governorate, (30° 33′ N, 31° 0′ E, 20 m a.s.l.), Egypt. The collection of plant material complies with the guidelines of the Ethics Committee in the National Research Centre. It was burned at 400 °C for 1 h. under nitrogen to get the biochar. After that, it was ground and sieved by a sieve with a size 160 mesh to get a very fine powder^30^. The obtained powder was washed with HNO_3_ (1 N) to remove all carbonate, then washed with distilled water till the pH of the washing water became neutral.

### Preparation of the adsorbed composites

The bio-composites were prepared by melt-blending 100 g of EVA in a twin-screw extruder (Haake Rheomex TW100, USA) at 100 °C and a rotation speed of 60 rpm^31^. After melting, 35 g of biochar or sugar beet residues were added and mixed for 10 min to form bio-composites C1 or C2, respectively. The mixture was collected and left for cooling, then cut into small pieces suitable for feeding into a stainless-steel pressure mould to make sheets with dimensions 8 × 10 cm and 0.2 mm thick. The moulding was done using pressure (5 MPa) at 120 °C for 5 min, then cooled to room temperature under pressure.

### Characterization

FT-IR spectra of the prepared samples were recorded in the range of 400–4000 cm^− 1^ on a Shimadzu 8400 S FT-IR Spectrophotometer. The surface morphology of as-prepared composites was analyzed using a scanning electron microscope (SEM; JSM 6360LV, JEOL/Noran). The microscope was attached to a dispersive energy spectrometer (EDX). The images were obtained using an accelerating voltage of 20 kV. The TGA analysis was performed using a Shimadzu TGA-50 thermogravimetric analyzer, Columbia, EUA, under nitrogen gas at 10 °C/min heating rate in the range from room temperature to 700 °C. While DSC was recorded on TA Instruments DSC Q20 V24, 11 Build 124. All samples were heated with a scan rate of 10 °C/min over a temperature range of 30–700 °C under a nitrogen atmosphere.

### Sorption study

#### Evaluation of Cu^2+^ and Pb^2+^ adsorption performance across synthetic composites

A comparative investigation was executed to evaluate the adsorption efficiency of two synthesized composites, identified as C1 and C2, for the removal of copper and lead ions from aqueous solutions. In each experimental run, a precise 50 mg dosage of the sorbent was integrated into 100 mL of a single-metal solution maintained at an initial concentration of 150 mg/L. To determine the influence of temporal dynamics on metal uptake, the mixtures were subjected to continuous agitation at ambient temperature for specific contact intervals of 30 and 60 min. Following the designated contact periods, the solid and liquid phases were separated through filtration. The residual concentration of the ions in the filtrate was quantified utilizing a Varian Spectra A220 atomic absorption spectrometer. The adsorption capacity (q), defined as the quantity of ions sequestered per unit mass of the composite material, was determined according to the following mass balance Eq. (1):

1 $$\:q=\left({C}_{0}-{C}_{t}\right)\times\:\frac{V}{M}$$

In this expression, C_0_ and C_t_ represent the initial and residual metal ion concentrations (mg/L), respectively. V denotes the total volume of the aqueous phase (L), while M corresponds to the dry mass of the sorbent employed (g).

#### Influence of contact duration on sorption kinetics

The kinetic behavior of copper (Cu^2+^) and lead (Pb^2+^) ions uptake onto the synthesized composites was investigated by varying the contact duration. To evaluate the impact of residence time on adsorption efficiency, experiments were performed across a temporal range of 5–120 min. For each experimental run, a standardized adsorbent dosage of 50 mg was introduced into an aqueous medium with an initial metal concentration of 150 mg/L. Following the specified intervals, the phase separation was conducted, and the adsorption capacity (q) was quantified according to the mass balance relationship defined in Eq. (1). This systematic approach allows for the identification of the equilibrium time and the determination of the rate-limiting steps governing the interaction between the metallic analytes and the composite surfaces.

#### Influence of adsorbent dosage on adsorption efficiency

The relationship between the mass of the synthesized composites and their metal-binding performance was evaluated by systematically varying the adsorbent dosage. To determine the optimal concentration of the solid phase, experiments were conducted using a range of sorbent masses: 50, 100, 200, 400, and 500 mg. These trials were performed using aqueous solutions containing Cu^2+^ or Pb^2+^ at a standardized initial concentration of 150 mg/L. To ensure the system reached a representative state of uptake, the contact durations were set based on preliminary kinetic data, with Cu^2+^ solutions filtered after 30 min and Pb^2+^ solutions after 60 min. Following the liquid-solid separation, the residual concentrations in the filtrate were quantified via Atomic Absorption Spectroscopy (AAS). The specific adsorption capacity (q) was subsequently determined using the mass balance relationship defined in Eq. (1).

#### Impact of pH on pollutant removal

To evaluate the critical influence of solution pH on the removal process of metals, batch adsorption experiments were conducted across a wide initial pH spectrum ranging from 2 to 9 using hydrochloric acid and sodium hydroxide. Solutions were prepared with an initial heavy metal concentration of 150 mg/L for both Cu^2+^ and Pb^2+^ ions. For each experimental run, a standardized adsorbent dosage of 50 mg of the composites (C1 or C2) was introduced into 100 mL of the metal ion solution. The suspensions were stirred at a constant temperature for a contact time 30 min for Cu^2+^ and 60 min for Pb^2+^, ensuring the system reached operational equilibrium.

#### Influence of initial metal concentration on adsorption capacity

The relationship between the initial solute concentration and the resultant uptake performance was evaluated by varying the concentrations of Cu^2+^ and Pb^2+^ across a range of 20, 40, 100, 160, and 200 mg/L. To maintain experimental consistency, the solution pH was stabilized at a constant pH of 6, which is often optimal for preventing metal precipitation. For each concentration gradient, a fixed adsorbent mass of 50 mg was utilized. The phase contact intervals were established at 30 min for Cu^2+^ and 60 min for Pb^2+^ to ensure the systems approached equilibrium.

#### Kinetic modeling and equilibrium isotherm investigations

To gain a deeper understanding of the adsorption mechanisms and the equilibrium dynamics between the ions and the sorbent surfaces, the experimental datasets were subjected to rigorous analysis using established theoretical models^32^ (detailed in Table S1).

Kinetic Models: The dynamics of ion removal were evaluated by fitting data to several linear and nonlinear kinetic models, including (pseudo-first-order^33^, pseudo-second-order^34^, intra-particle diffusion^35^, and Elovich^36^.

Adsorption Isotherms: The equilibrium interaction between the ions and the sorbent surface was described using linear and nonlinear (Langmuir^37^, Freundlich^38^, Temkin^38^, and The Dubinin–Radushkevich (D–R)^39^ models were used to study the kinetics of ions removal and the interaction between ions and the sorbent, respectively.

#### Error analysis and model validation

To ensure validation and avoid the inherent bias of linearized R^2^ values, all kinetic and isotherm fits were evaluated using non-linear mathematical optimization. Initial residual analysis confirmed the absence of systematic error between the experimental (q_exp_) and theoretically predicted (q_cal_) adsorption capacities. The overall goodness-of-fit was subsequently quantified using three standard error functions: the Sum of Squared Errors (SSE), Root Mean Squared Error (RMSE), and the non-linear Chi-square ($\:{\chi\:}^{2}$) test. A minimization of these error values indicates a superior representation of the actual adsorption mechanism. The respective equations are defined as follows^40^:

2 $$\:\mathrm{S}\mathrm{u}\mathrm{m}\:\mathrm{o}\mathrm{f}\:\mathrm{S}\mathrm{q}\mathrm{u}\mathrm{a}\mathrm{r}\mathrm{e}\mathrm{d}\:\mathrm{E}\mathrm{r}\mathrm{r}\mathrm{o}\mathrm{r}\mathrm{s}\:\left(\mathrm{S}\mathrm{S}\mathrm{E}\right):\sum\limits_{i=1}^{n}{\left({q}_{exp}-{q}_{cal}\right)}^{2}$$

3 $$\:\mathrm{R}\mathrm{o}\mathrm{o}\mathrm{t}\:\mathrm{M}\mathrm{e}\mathrm{a}\mathrm{n}\:\mathrm{S}\mathrm{q}\mathrm{u}\mathrm{a}\mathrm{r}\mathrm{e}\mathrm{d}\:\mathrm{E}\mathrm{r}\mathrm{r}\mathrm{o}\mathrm{r}\:\left(\mathrm{R}\mathrm{M}\mathrm{S}\mathrm{E}\right):\sqrt{\frac{1}{n}\sum\limits_{i=1}^{n}{\left({q}_{exp}-{q}_{cal}\right)}^{2}}$$

4 $$\:\mathrm{N}\mathrm{o}\mathrm{n}-\mathrm{l}\mathrm{i}\mathrm{n}\mathrm{e}\mathrm{a}\mathrm{r}\:\mathrm{C}\mathrm{h}\mathrm{i}-\mathrm{s}\mathrm{q}\mathrm{u}\mathrm{a}\mathrm{r}\mathrm{e}\:\left({\chi\:}^{2}\right):\sum\limits_{i=1}^{n}\frac{{\left({q}_{exp}-{q}_{cal}\right)}^{2}}{{q}_{cal}}$$

where n represents the number of experimental data points, q_exp_ is the experimentally measured capacity, and q_cal_ is the capacity predicted by the corresponding theoretical model.

## Results and discussion

### Physicochemical characterization

**Fig. 1:**
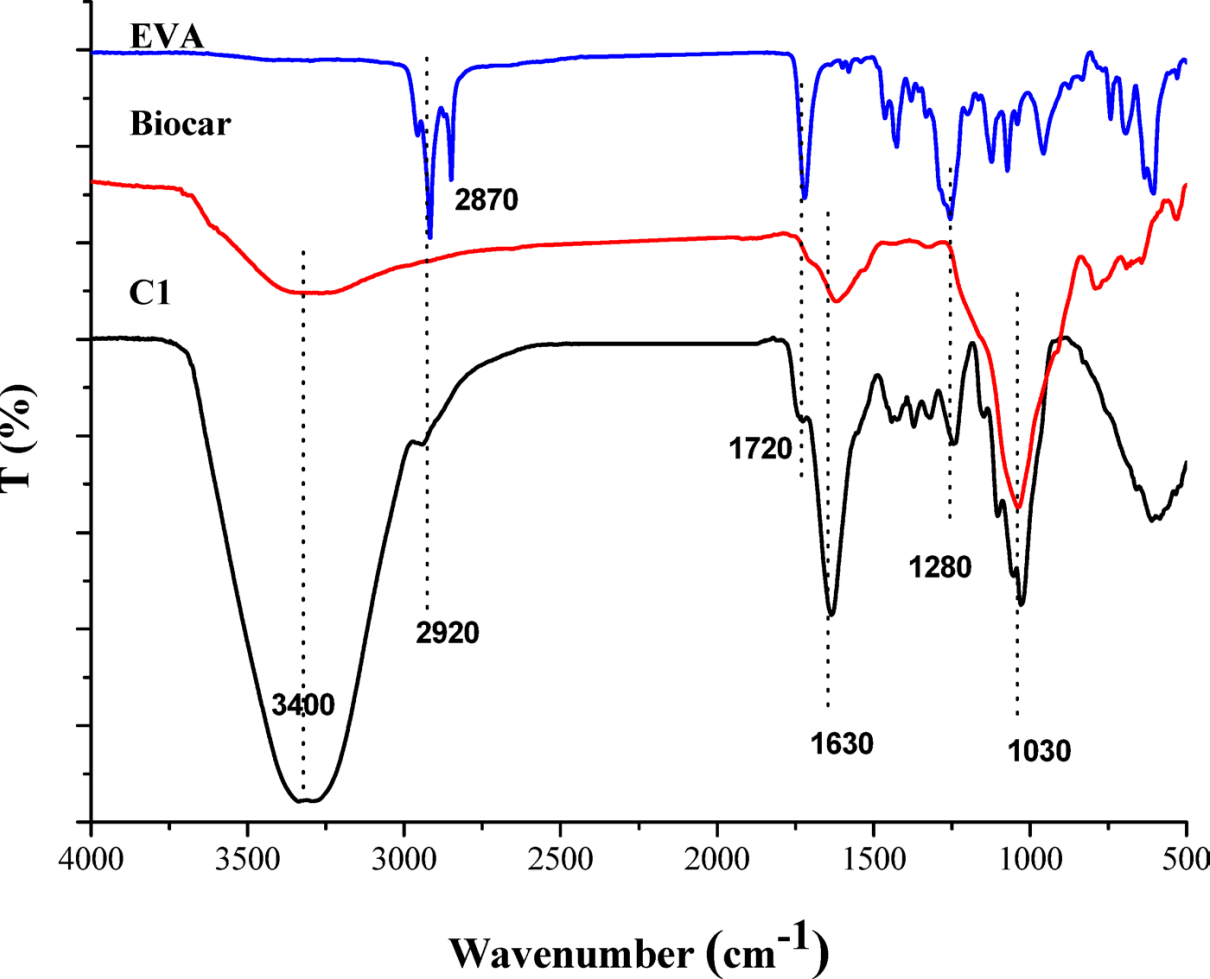
FTIR spectra of pure EVA and bio-composite (C1) compared with the pure biochar.

Figure 1 represents the FTIR spectra of pure EVA, biochar, and EVA/biochar bio-composite (C1). The FTIR analysis of pure biochar exhibits a wide absorption peak around 3350 cm^−1^, assigned to O–H stretching from phenolic groups, residual water, and surface oxygen functionalities formed via pyrolysis. Peaks around 1630 cm^−1^ correspond to aromatic C=C stretch and 1030 cm^−1^ attributed to C–O stretch of alcohols, phenols, and ethers groups, indicating partial carbonization of its lignocellulosic components^41–43^.

Pure EVA shows typical copolymer bands: strong C–H aliphatic stretches at 2850–2920 cm^−1^ and a prominent C=O stretch from vinyl acetate at 1735 cm^[−144^. In the biochar/EVA bio-composite (C1), major bands from both materials persist post-melt blending, with no major chemical changes, though –OH and C=O regions exhibit intensity shifts and minor displacements referring to H-bonding and dipole interactions between biochar’s oxygen groups and EVA’s polar segments. Increased C–H band intensities and retained oxygenated groups further evidence uniform distribution of biochar in EVA, along with strong filler-polymer interfacial adhesion. These interactions boost the structural stability, polarity, and adsorptive capacity of the prepared bio-composite.

**Fig. 2:**
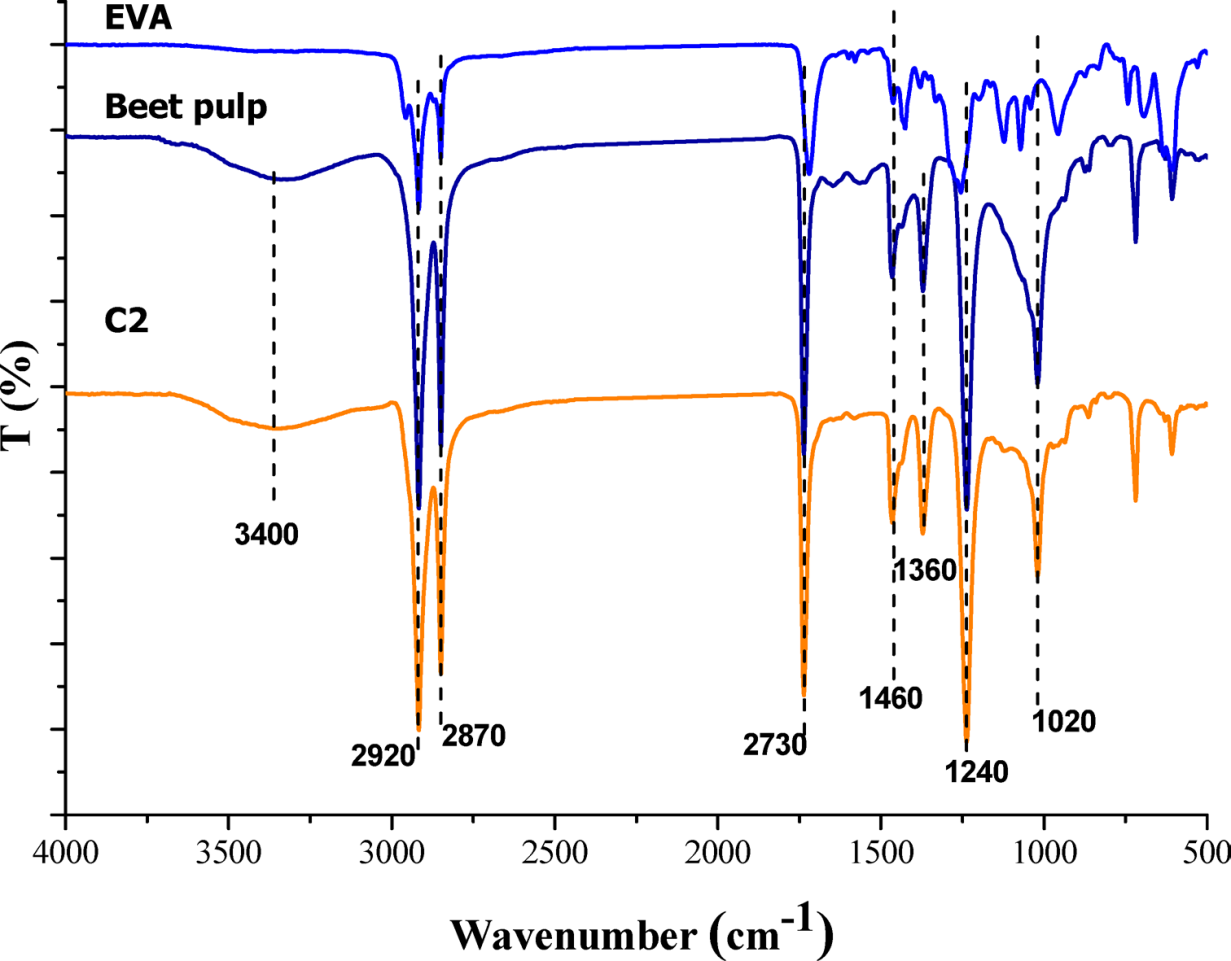
FTIR spectra of pure EVA and bio-composite (C2) in compared with the beet pulp waste.

FTIR characterization of pure EVA, sugar beet pulp waste, and EVA/sugar beet pulp waste bio-composite (C2) is shown in Fig. 2. The FTIR spectra confirm the effective integration of lignocellulosic sugar beet pulp into the EVA matrix, maintaining key chemical groups essential for adsorption. The spectrum of the pure beet pulp waste exhibits a broad absorption band around 3400 cm^−1^, which is attributed to the stretching vibrations of hydroxyl (–OH) groups found in cellulose, hemicellulose, and residual moisture. The bands observed in the region of 2920 –2870 cm^−1^ are related to the stretching vibrations of aliphatic C–H groups. The characteristic peak at 1730 cm^−1^ is assigned to the carbonyl (C=O) groups stretching vibrations, mainly associated with ester and carboxylic functionalities. Furthermore, C–C, C–O, and C–O–C stretching vibrations inside the polysaccharide backbone of the lignocellulosic structure are associated with the strong bands in the region between 1460 and 900 cm^[− 145^. The resulting bio-composite (C2) retains the major absorption bands of both components post-melt blending, with minor shifts/intensity changes in O–H/C=O regions that refer to H-bonding and dipole interactions between pulp hydroxyls and EVA acetate. Ester band overlap implies strong interfacial adhesion and uniform filler dispersion, preserving adsorption-active groups for Cu^2+^/Pb^2+^ binding. Thus, the EVA/beet pulp bio-composite offers a stable, active adsorbent for wastewater remediation.

The adsorption capacity of the prepared EVA-based bio-composites is mainly governed by the presence of oxygen-containing functional groups and porous surface structures of the prepared bio-composite produced from biochar and sugar beet pulp fillers. FTIR analysis revealed several peaks that were predicted to be active adsorption sites for metal ions. The presence of –OH, –C=O, C–O, and C–O–C groups plays a major role in the formation of hydrogen bonds and metal-ion complexation^46–48^.

**Fig. 3:**
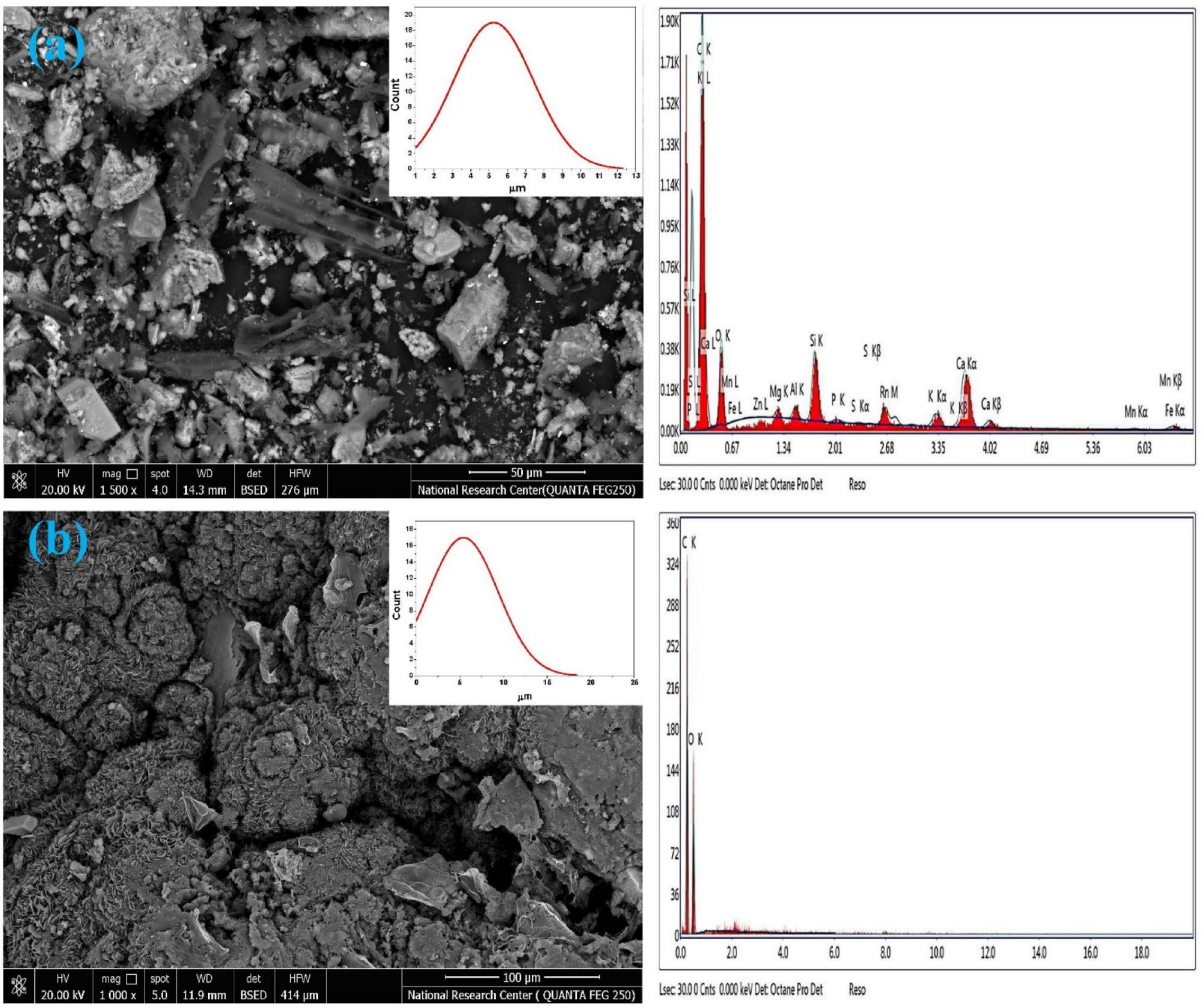
SEM image of biochar (**a**; x = 1500) and sugar beet pulp waste (**b**; x = 1000), and their corresponding EDX.

Figure 3a shows the SEM micrograph of the prepared biochar and revealing a highly uneven and porous surface morphology with fine particles primarily smaller than 50 μm. Particle size distribution was determined from the SEM micrograph shown in Fig. 3a using image analysis software. The resulting histogram indicated that the mean particle size was about 5 μm. The thermal treatment during pyrolysis, which encourages the formation of a porous carbonaceous structure, is responsible for the abundance of cavities, fissures, and rough surface features. These morphological features are beneficial for adsorption applications. Carbon is the main component, followed by oxygen and some inorganic elements, including Si, Al, Ca, Mg, Fe, Mn, Zn, P, and S, according to the related EDX spectrum^30^. These mineral elements are present in the biomass precursor and may facilitate adsorption through surface complexation or ion exchange. These elements enhance adsorption by adding inorganic active sites to the bio-composite surface. Ca^2+^ and Mg^2+^ enable ion exchange with Cu^2+^ and Pb^2+^ from solution, while Fe/Al oxides facilitate surface complexation via coordination with oxygen groups. Silica domains boost surface polarity and site accessibility, improving efficiency^49–51^.

Figure 3b shows the SEM image of sugar beet pulp waste, which exhibits an irregular, fibrous, and heterogeneous surface structure reflecting the presence of cellulose, hemicellulose, and residual organic matter. Particle size distribution was carried out from the SEM micrograph of sugar beet pulp waste (Fig. 3b) using image analysis software. The obtained histogram revealed that the average particle size was 6.5 μm. The EDX analysis shows that carbon and oxygen are the predominant constituents, confirming the organic nature of the beet pulp waste.

**Fig. 4:**
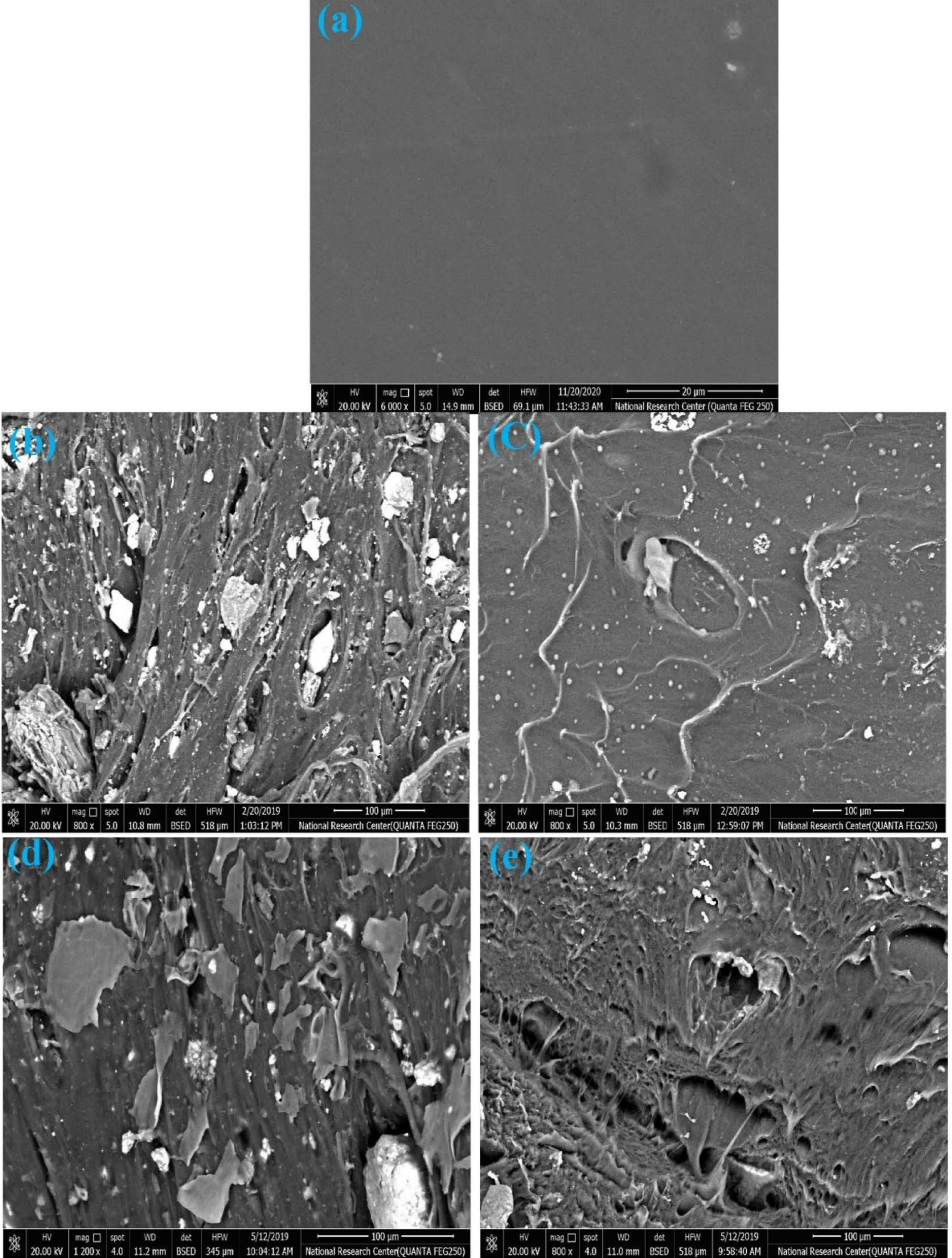
SEM image of pure EVA (**a**) and bio-composite C1 and C2 (**b** and **c**, respectively) before ions adsorption and (**d** and **e**, respectively) after ion adsorption.

Figure 4 shows the SEM micrographs of pure EVA and the prepared bio-composites C1 and C2 before and after metal ion adsorption. Pure EVA (Fig. 4a) shows a smooth, compact, and fairly uniform surface, which is typical of the nature of the thermoplastic matrix. This morphology suggests a lack of sufficient accessible active sites, thereby accounting for the poor adsorption performance of pure EVA toward heavy metal ions. Before ion adsorption, the surface of bio-composite C1 (Fig. 4b), which has biochar dispersed throughout the EVA matrix, is comparatively rough and heterogeneous, with irregular cavities and well-distributed porous domains. These characteristics show good interfacial compatibility with the polymer matrix and are derived from the biochar particles. Because they increase the accessible surface area and offer active sites for metal ion binding, such surface roughness and porosity are advantageous for adsorption. On the other hand, bio-composite C2 (Fig. 4c), which incorporates sugar beet pulp waste, exhibits a less compact and more fibrous shape with irregular agglomerates and linked voids. This structure indicates the existence of several functional groups exposed at the surface and represents the lignocellulosic nature of the beet pulp.

Both bio-composites exhibit discernible morphological changes during ion adsorption (Fig. 4d and e). Following adsorption, the surface of C1 seems denser and largely covered (Fig. 4d), with fewer apparent pores and voids. This indicates successful ion uptake and can be explained by metal ions occupying adsorption sites and accumulating inside the porous biochar domains. Similar surface smoothing and partial pore blocking are seen in bio-composite C2 following adsorption (Fig. 4e), along with the formation of deposits or clusters that are probably related to adsorbed metal ions interacting with hydroxyl, carboxyl, and other oxygen-containing functional groups found in the beet pulp waste. The SEM data show that both bio-composites undergo unique surface changes during adsorption, providing evidence of metal ion adsorption. The more pores seen for C1 compared to C2 are consistent with its higher adsorption capacity, as discussed in the adsorption and kinetic studies. These findings verify that the adsorption performance of EVA-based bio-composites is significantly influenced by surface morphology and filler type.

**Fig. 5:**
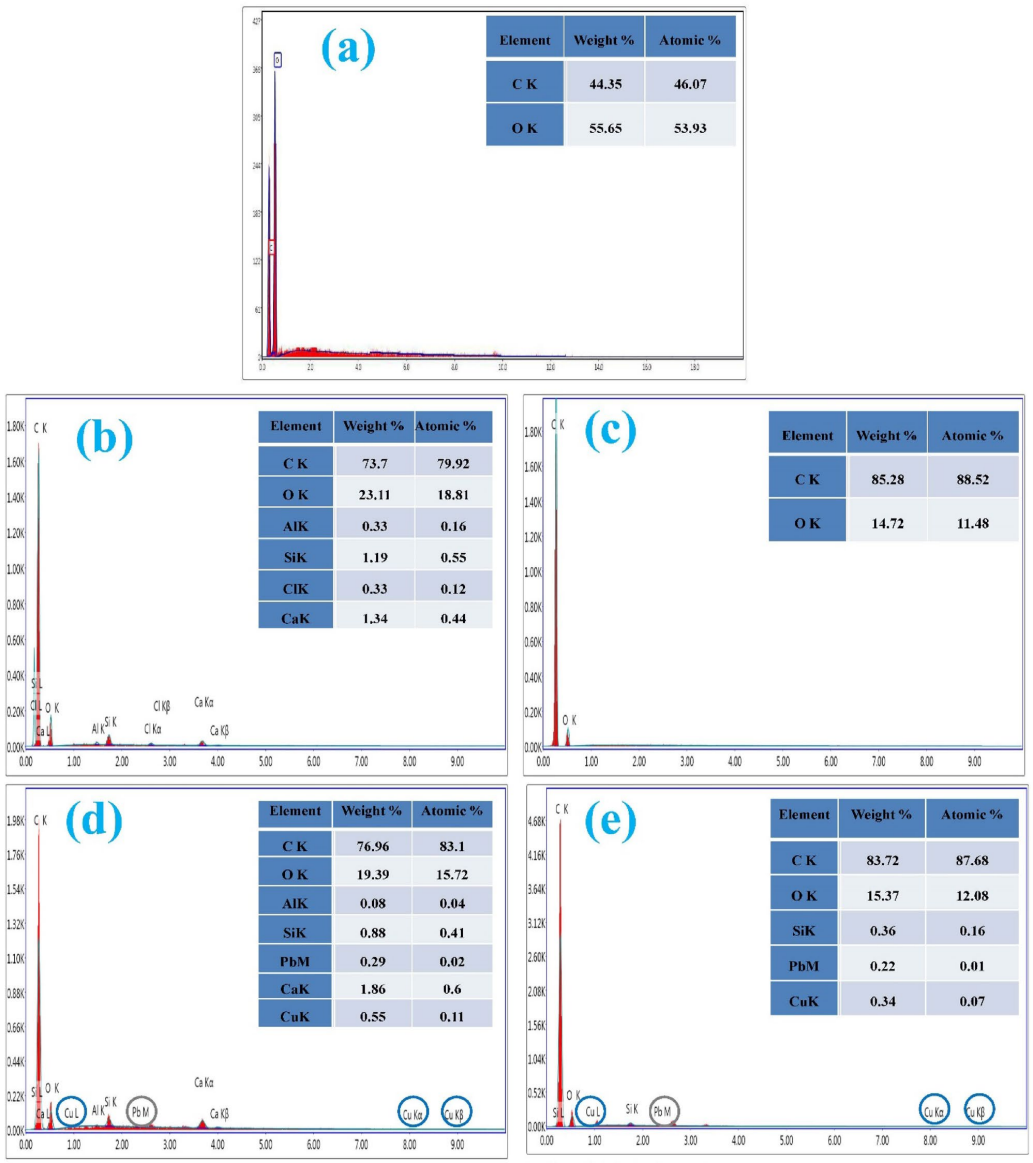
EDX image of pure EVA (**a**) and bio-composite C1 and C2 (**b** and **c**, respectively) before ions adsorption and (**d** and **e**, respectively) after ion adsorption.

Figure 5 illustrates the EDX spectra of bio-composites C1 and C2 before (Fig. 5a, b) and after metal ion adsorption (Fig. 5c, d). Before ion adsorption, the EDX spectrum of bio-composite C1 shows signals of carbon and oxygen from the EVA matrix and biochar filler (Fig. 5a), with smaller peaks representing inorganic elements like Si, Al, Ca, Mg, and Fe, which indicate that the biochar was successfully integrated into the polymer matrix. Similar to the lignocellulosic sugar beet pulp waste, bio-composite C2 (Fig. 5b) exhibits high carbon and oxygen peaks.

The presence of new peaks corresponding to Cu²⁺ and/or Pb²⁺ ions in the EDX spectra of both bio-composites (Fig. 5c, d) confirmed the successful adsorption of metal ions on the composite surfaces. The appearance of lead and copper peaks in bio-composite C1 (0.29 and 0.55%, respectively) with high content compared to C2 (0.22 and 0.34%, respectively) indicates that higher adsorption and interaction between metal ions and functional groups on the surface of the biochar, compared to sugar beet pulp.

**Fig. 6:**
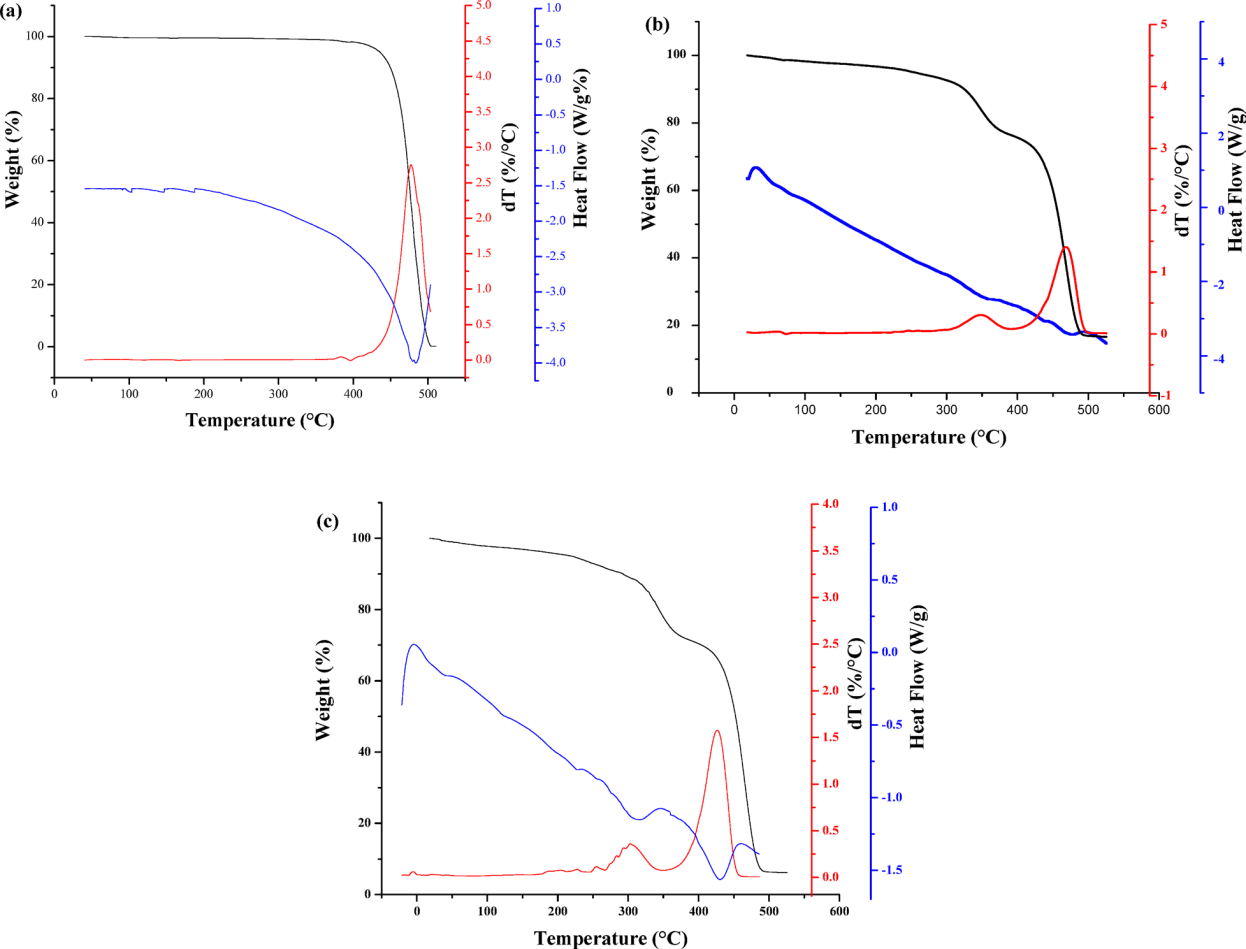
TG and DSC of pure EVA (**a**) and the bio-composite C1 (**b**) and C2 (**c**).

The thermogravimetric (TG) and differential scanning calorimetry (DSC) curves of pure EVA and the bio-composites C1 and C2 are illustrated in Fig. 6. The TG profile shows that EVA exhibits good thermal stability at low temperatures, with negligible weight loss below ~ 150 °C attributed to residual moisture. The major degradation occurs in two successive stages: the first (300–380 °C) involves deacetylation of vinyl acetate groups releasing acetic acid, while the second (400–450 °C) corresponds to polyethylene segment decomposition and carbonaceous structure breakdown (Fig. 6a). The DSC thermogram displays a characteristic endothermic melting peak from crystalline polyethylene domains, with a single, well-defined transition indicating a homogeneous polymer phase and confirming EVA’s semi-crystalline nature. This peak reflects an orderly chain arrangement in crystalline regions, while the absence of additional transitions confirms no significant secondary phases or impurities (Fig. 6a).

The elimination of physically adsorbed moisture and volatile components linked to the biochar is responsible for the initial weight loss shown in the TG curve of bio-composite C1 (Fig. 6b) below 120 °C. The primary thermal degradation takes place between 300 and 450 °C, which corresponds to the breakdown of organic functional groups on the surface of the biochar and the disintegration of the EVA matrix. Because the filler is inorganic and carbonaceous, the introduction of biochar results in a greater residual mass at higher temperatures, indicating improved thermal stability and char formation.

Bio-composite C2 (Fig. 6c) exhibits a more noticeable initial weight loss below 150 °C, which is a result of moisture evaporation and the thermal breakdown of labile components in the sugar beet pulp waste. Because the lignocellulosic filler has less thermal stability than in C1, the principal degradation process occurs at somewhat lower temperatures. The breakdown of cellulose, hemicellulose, and EVA matrix is represented by the subsequent mass loss between 250 and 400 °C. As a result, at high temperatures, C2 shows less residual mass than C1.

The DSC thermograms further support these observations. The DSC curve for bio-composite C1 (Fig. 6b) displays a distinctive melting endotherm of the EVA matrix with a comparatively sharp and well-defined peak, indicating good biochar dispersion and little disruption of the polymer crystalline areas. As a result of the lignocellulosic filler’s presence and interaction with the EVA matrix, bio-composite C2 (Fig. 6c) exhibits a larger melting endotherm, indicating decreased crystallinity and greater chain mobility.

### Sorption study

#### Evaluation of Cu²⁺ and Pb²⁺ adsorption performance across synthetic composites

**Fig. 7:**
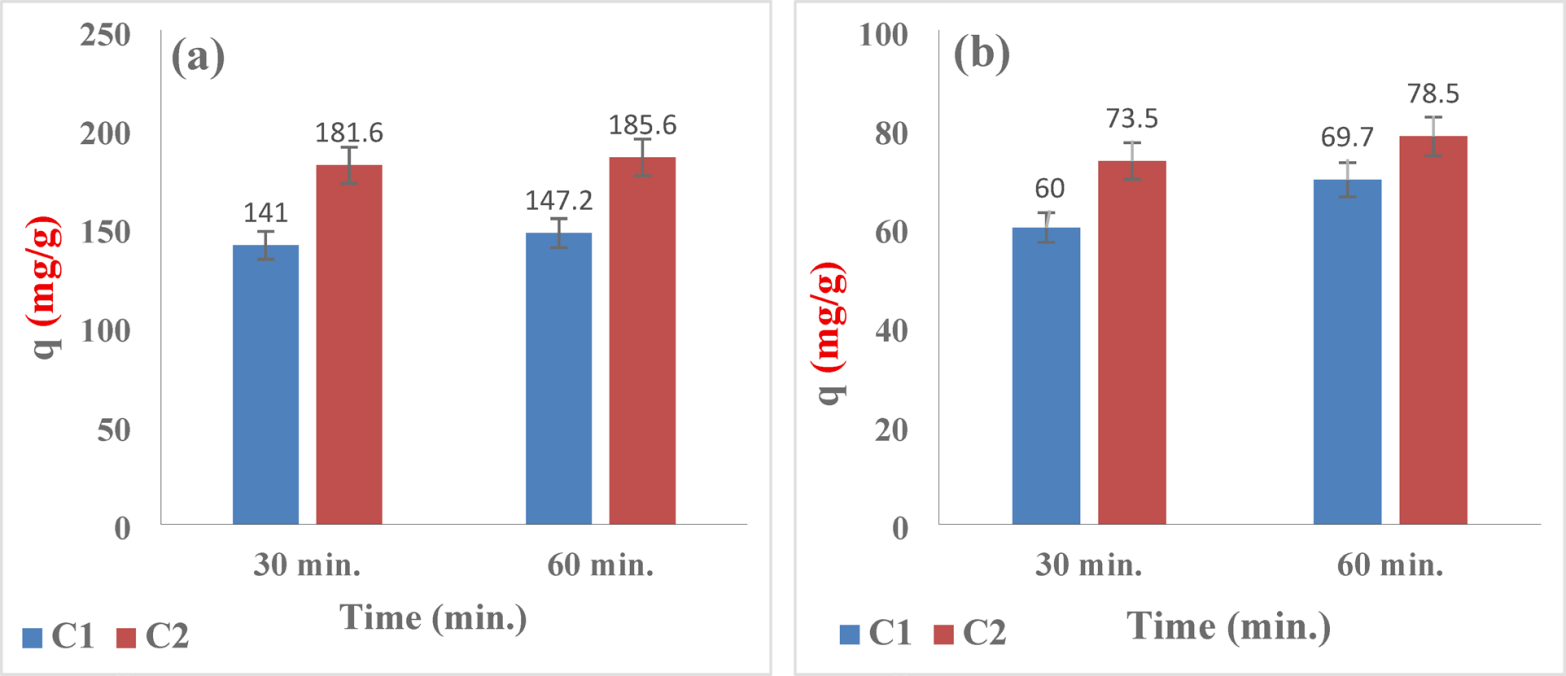
Comparative adsorption capacities for (**a**) Cu^2+^ and (**b**) Pb^2+^ utilizing the synthesized sorbents at contact intervals of 30 and 60 min. The experimental parameters were maintained at a standardized adsorbent dosage of 50 mg in 100 mL of aqueous solution, with an initial concentration of 150 mg/L for both metals’ ions.

The adsorptive efficacy of two distinct synthesized materials, designated as C1 and C2, was rigorously evaluated for the adsorption of divalent copper and lead ions from aqueous media. Experimental trials were conducted using a standardized adsorbent dosage of 50 mg in 100 mL of ions solution with an initial concentration of 150 mg/L. The temporal influence on uptake was monitored at intervals of 30 and 60 min. As illustrated in Fig. 7, both composites demonstrated significant capacity for heavy metals removal, with specific performance variations observed between the two sorbent types and the targeted metal species. Analysis of the results reveals that C2 exhibited a superior adsorption capacity (q) for Cu^2+^ compared to C1. Specifically, C2 achieved a high initial uptake of 181.6 mg/g in 30 min, which increased slightly to 185.6 mg/g at 60 min. In contrast, C1 recorded an adsorption capacity of 141.0 mg/g after 30 min, reaching 147.2 mg/g by the 60 min. A similar performance trend was observed during the adsorption of Pb^2+^, although the absolute values for adsorption capacity were notably lower than those recorded for copper. C2 maintained a higher efficiency, with capacities of 73.5 mg/g and 78.5 mg/g at 30 and 60 min, respectively. C1 exhibited an uptake of 60.0 mg/g at 30 min, which rose to 69.7 mg/g after 60 min. The results indicate that C2 consistently outperforms C1 in the removal of both metals ions under the tested conditions. The consistently higher performance of biomass precursor contains inherent mineral elements, such as Ca^2+^, Mg^2+^, and K^+^, which play a significant role in the remediation process. These minerals facilitate adsorption through ion exchange, where the divalent heavy metal cations displace the lighter alkali/alkaline earth metals within the bio-composite framework. Furthermore, these sites contribute to surface complexation, acting as coordination centers that stabilize the attachment of metals to the oxygen-containing functional groups (e.g., carboxyl and hydroxyl groups) present in the EVA-hybrid matrix^52^. This superiority likely stems from a higher density of surface-active sites or a more favorable pore structure within C2 composite.

#### Influence of contact duration on sorption kinetics

**Fig. 8:**
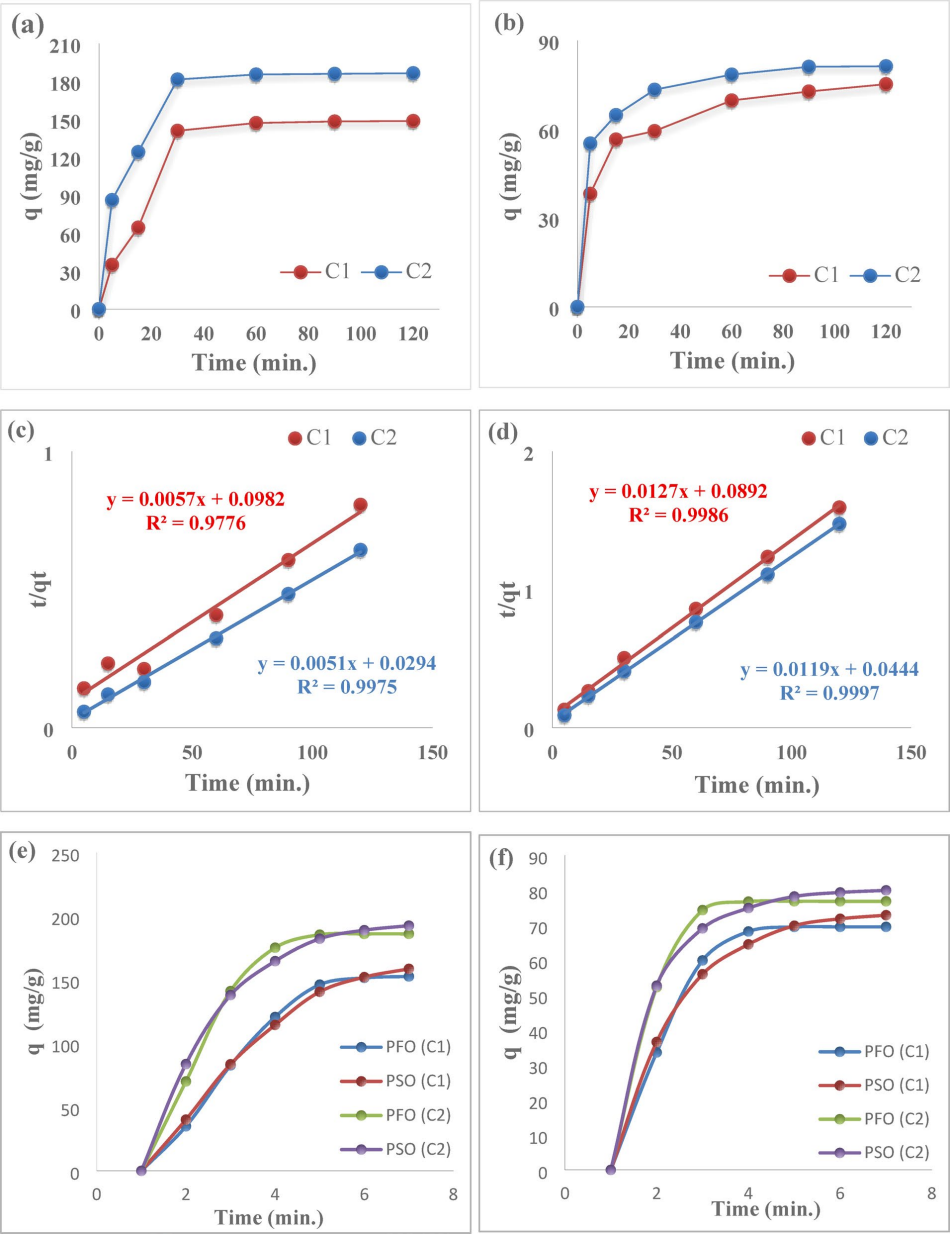
Temporal influence of contact time on the uptake capacity of (**a**) Cu^2+^ and (**b**) Pb^2+^ by sorbents C1 & C2, alongside the corresponding linearized pseudo-second-order kinetic models for (**c**) Cu^2+^ and (**d**) Pb^2+^, and the non-linear pseudo-first-order (PFO) and pseudo-second-order (PSO) kinetic regressions fitted to (**e**) Cu^2+^ and (**f**) Pb^2+^ adsorption data. [Experimental parameters: sorbent dosage = 50 mg, solution volume = 100 mL, and initial metal concentration = 150 mg/L].

The temporal dynamics of copper and lead ions adsorption onto the synthesized composites, C1 and C2, were evaluated to determine the equilibrium parameters and the rate of pollutant removal. To maintain experimental consistency, a standardized dosage of 50 mg of each sorbent was introduced into 100 mL of an aqueous solution containing an initial metal concentration of 150 mg/L at ambient temperature. As illustrated in Figs. 8a and b, both species exhibited a biphasic adsorption profile characterized by an initially rapid adsorption rate followed by a gradual deceleration as the system approached equilibrium. For Cu^2+^, a significant portion of the total uptake was achieved within the first 30 min, whereas Pb^2+^ required a longer interval of approximately 60 min to reach a similar state of site occupancy. This rapid initial phase is largely attributed to the high density of unoccupied active binding sites on the sorbent surfaces, which facilitates immediate interaction with the metal ions. As the contact time progressed, the rate of adsorption diminished significantly; this decline in uptake velocity is characteristic of the decreasing availability of vacant sites as the surface becomes saturated^53^. The maximum adsorption capacities recorded during the equilibrium phase were found to be metal-specific and sorbent-dependent. For Cu^2+^, C1 and C2 reached maximum capacities of 148 mg/g and 186 mg/g, respectively. In the case of Pb^2+^, the equilibrium capacities were notably lower, measured at 75 mg/g for C1 and 81 mg/g for C2. These quantitative results indicate that while both materials are effective for heavy metal remediation, the C2 composite demonstrates superior performance, and the affinity for copper is consistently higher than that for lead across both substrates.

To gain further insights into the adsorption mechanism, the linear and non-linear kinetic behavior was comprehensively evaluated by comparing the fitness of various models. To avoid potential mathematical distortions and parameter bias induced by linear transformations, model suitability was rigorously cross-examined using absolute error functions namely, the Sum of Squared Errors (SSE) and Chi-square$\:{\:(\chi\:}^{2})$ alongside the conventional coefficient of determination (R^2^). As shown in Table 1, analysis of the Cu^2+^ data reveals a notable discrepancy between the two regression methodologies. When assessing the linearized plots, the pseudo-second order (PSO) model initially appeared to be the undisputed descriptor for both sorbents, yielding exceptionally high linear correlation coefficients (R^2^ = 0.9776 for C1 and R^2^ = 0.9975 for C2). The calculated linear PSO capacity for Cu^2+^ on C2 was 196 mg/g, closely matching the experimental 186.08 mg/g. However, non-linear optimization revealed more nuanced behavior; for the C1 composite, the non-linear pseudo-first order (PFO) expression unexpectedly exhibited a lower error distribution ($\:{\chi\:}^{2}$= 7.65) than the non-linear PSO framework ($\:{\chi\:}^{2}$= 12.21). Furthermore, non-linear regression successfully resolved a severe mathematical artifact inherent to the linearized PFO model for C2, which had artificially compressed the calculated capacity to an unrealistic 56.80 mg/g; the non-linear PFO accurately corrected this parameter to 186.1 mg/g, converging closely with the non-linear PSO fit (SSE = 538.9, $\:{\chi\:}^{2}$= 3.49). In contrast as shown in Table 2, the kinetic profile of Pb^2+^ capture presented a highly robust and cohesive fit across both regression styles. The linearized PSO model yielded near-perfect alignment (R^2^ > 0.9986), with calculated linear values of 78.7 mg/g (C1) and 84 mg/g (C2) matching the experimental observations of 75.8 mg/g and 81.1 mg/g, respectively. This selection was strongly vindicated by non-linear optimization, where the PSO model displayed heavily minimized error boundaries (SSE = 35.8, $\:{\chi\:}^{2}$= 0.57 for C1; and SSE = 31.9, $\:{\chi\:}^{2}$= 0.48 for C2) along with an excellent convergence of non-linear capacities (q_e_, (cal) = 76.16 mg/g for C1, and 81.8 mg/g for C2). Collectively, these findings indicate that the adsorption rate is intricately governed by the availability of vacant active binding sites and the strength of the surface interactions, safely confirming that chemical adsorption involving valence forces through the sharing or exchange of electrons serves as the predominant rate-limiting step^40,54^.

To further corroborate this chemisorption mechanism on the energetically heterogeneous composite surfaces, the Elovich model was evaluated, where its mathematical correlation reflects the structural heterogeneity across the active binding sites. This is robustly substantiated by the minimized non-linear error profiles observed for the Pb^2+^ system (SSE = 23.9, $\:{\chi\:}^{2}$= 0.46 for C1; and SSE = 10.3, $\:{\chi\:}^{2}$= 0.14 for C2). The high initial adsorption rate constants (α) paired with low desorption constants (β) point toward a highly stable chemical immobilization pathway that fully supports the pseudo-second-order conclusions. In contrast, the Cu^2+^ kinetic profiles exhibited noticeably higher error distributions under the Elovich framework $\:({\chi\:}^{2}$= 18.93 for C1, and $\:{\chi\:}^{2}$= 7.65 for C2), statistically verifying that the active sites involved in copper binding operate under a more uniform, less energetically diverse environment, which correlates seamlessly with its monolayer Langmuir equilibrium behavior^55^. Concurrently, to isolate the specific diffusion barriers, the Intra-particle Diffusion model was calculated (See, Tables 1 and 2). This model yielded linear correlation coefficients (R^2^ = 0.73–0.74 for Cu^2+^ and R^2^ approx. 0.89 for Pb^2+^) that were significantly inferior to the pseudo-second-order kinetics, a trend further verified by exceptionally high non-linear error distributions. Because these fits are mathematically poor and the intercept values (C) do not pass through the origin, intra-particle pore diffusion cannot be defined as the sole rate-limiting barrier. Instead, the large, non-zero intercept constants (C = 27.2–85.9 mg/g for Cu^2+^ and 36.2–52.9 mg/g for Pb^2+^) reflect a substantial boundary layer thickness, conclusively proving that external film diffusion resistance operates concurrently with pore migration and surface chemisorption within a multi-step rate-controlling mechanism^49,56^.

**Table 1:** Linear and nonlinear kinetic parameters for the adsorption of Cu^2+^ utilizing the synthesized composites C1 and C2.

Kinetics models constants													
Pseudo-first-order	K_1_ (min^-1^)		q_e_ (exp.) (mg/g)		q_e_ (cal.) (mg/g)			R^2^			SSE	RMSE	Chi-square
	Linear	Nonlinear	Linear	Nonlinear	Linear		Nonlinear	Linear		Nonlinear	Nonlinear	Nonlinear	Nonlinear
C1	0.0875	0.05	148.4	148.4	188.8		152.9	0.9512		0.9402	773.52	11.35	7.65
C2	0.3799	0.0947	186.08	186.08	56.80		186.1	0.7692		0.9366	584.1	9.87	5.92

Pseudo-second-order	K_2_ (g/mg min)		q_e_ (exp.) (mg/g)		q_e_ (cal.) (mg/g)			R^2^			SSE	RMSE	Chi-square
	Linear	Nonlinear	Linear	Nonlinear	Linear	Nonlinear		Linear		Nonlinear	Nonlinear	Nonlinear	Nonlinear
C1	0.0003	0.0003	148.4	148.4	175.4	181.8		0.9776		0.9034	1249	14.43	12.21
C2	0.0008	0.00068	186.08	186.1	196	204		0.9975		0.9415	538.9	9.48	3.49

Intra-particle diffusion model	Kp (mg/ g min^1/2^)		C					R^2^			SSE	RMSE	Chi-square
	Linear	Nonlinear	Linear		Nonlinear			Linear		Nonlinear	Nonlinear	Nonlinear	Nonlinear
C1	13.1	13.1	27.2		27.3			0.7487		0.7487	3247.9	23.27	33.9
C2	10.93	10.93	85.9		85.9			0.7305		0.7305	2484	20.35	17.37

Elovich	α (mg/g min)		β (mg/ g min)					R^2^			SSE	RMSE	Chi-square
	Linear	Nonlinear	Linear		Nonlinear			Linear	Nonlinear		Nonlinear	Nonlinear	Nonlinear
C1	73.6	16.55	0.025		0.023			0.8695	0.8529		1900	17.79	18.93
C2	19.9	108.9	0.019		0.0297			0.8754	0.8724		1176	14	7.65

**Table 2:** Linear and nonlinear kinetic parameters for the adsorption of Pb^2+^ utilizing the synthesized composites C1 and C2.

Kinetics models constants														
Pseudo-first-order	K_1_ (min^-1^)		q_e_ (exp.) (mg/g)		q_e_ (cal.) (mg/g)			R^2^				SSE	RMSE	Chi-square
	Linear	Nonlinear	Linear	Nonlinear	Linear		Nonlinear	Linear	Nonlinear			Nonlinear	Nonlinear	Nonlinear
C1	0.034	0.1319	75.8	75.8	42.3		69.5	0.9696	0.8391			153.6	5.06	2.59
C2	0.04	0.23	81.16	81.16	30.15		76.8	0.9937	0.7223			151.8	5.03	2.07

Pseudo-second-order	K_2_ (g/mg min)		q_e_ (exp.) (mg/g)		q_e_ (cal.) (mg/g)			R^2^				SSE	RMSE	Chi-square
	Linear	Nonlinear	Linear	Nonlinear	Linear	Nonlinear		Linear			Nonlinear	Nonlinear	Nonlinear	Nonlinear
C1	0.002	0.0024	75.8	75.8	78.74	76.16		0.9986			0.9625	35.8	2.44	0.57
C2	0.003	0.004	81.16	81.16	84	81.8		0.9997			0.9415	31.9	2.3	0.48

Intra-particle diffusion model	Kp (mg/ g min^1/2^)		C					R^2^				SSE	RMSE	Chi-square
	Linear	Nonlinear	Linear		Nonlinear			Linear		Nonlinear		Nonlinear	Nonlinear	Nonlinear
C1	3.89	3.89	36.2		36.2			0.8943		0.8943		100.9	4.1	1.95
C2	2.93	2.9	52.9		52.9			0.8903		0.8903		59.9	3.2	0.86

Elovich	α (mg/g min)		β (mg/ g min)					R^2^				SSE	RMSE	Chi-square
	Linear	Nonlinear	Linear		Nonlinear			Linear		Nonlinear		Nonlinear	Nonlinear	Nonlinear
C1	79.2	76.6	0.088		0.087			0.9757		0.9749		23.9	1.99	0.46
C2	1139	1136.8	0.11		0.12			0.9812		0.9812		10.3	1.3	0.14

#### Influence of adsorbent dosage on adsorption efficiency

**Fig. 9:**
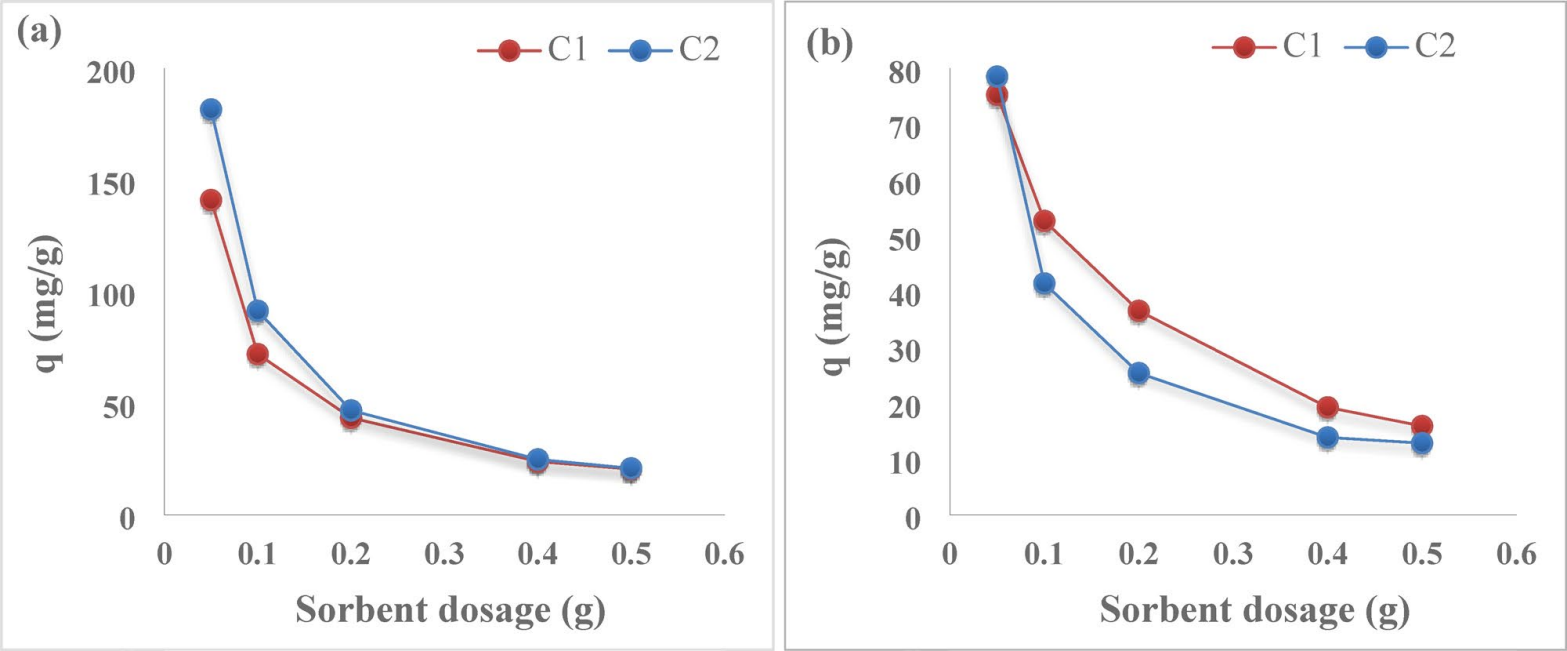
Variation in adsorption capacity as a function of adsorbent dosage for (**a**) Cu^2+^ and (**b**) Pb^2+^ utilizing bio-composites C1 and C2. The experiments were conducted with fixed contact intervals of 30 min for Cu^2+^and 60 min for Pb^2+^ within a 100 mL aqueous solution, initiated at a concentration of 150 mg/L for both ions’ species.

The mass of the adsorbent significantly influences the overall efficacy of the adsorption process, as the concentration of solid-phase active sites determines the total available surface for ion interaction. To evaluate this relationship, experiments were conducted by varying the sorbent dosage from 50 to 500 mg while maintaining a constant solution pH, contact time, and an initial metal concentration of 150 mg/L. The experimental results (Fig. 9) revealed a pronounced negative correlation between the adsorbent mass and the specific uptake capacity (q) for both Cu^2+^ and Pb^2+^ Specifically, for copper adsorption using C1, the adsorption capacity declined from 141 to 20 mg/g, while C2 exhibited a decrease from 181 to 21 mg/g as the dosage increased to the maximum tested value of 500 mg. A similar trend was observed for Pb^2+^, with C1 and C2 capacities dropping from 75 to 16 mg/g and 78.5 to 13 mg/g, respectively. This phenomenon is primarily attributed to the aggregation of adsorbent particles at higher solid-to-liquid ratios. Such agglomeration leads to a significant decrease in the total effective surface area and the shielding of active binding sites, rendering a portion of the material inaccessible to the metallic analytes^57^. The observed decrease in adsorption capacity (q) as sorbent dosage increases can be further elucidated through the mechanics of site saturation and concentration gradients. At lower adsorbent dosages, the high ratio of ions to available surface area ensures that the concentration of Cu^2+^ and Pb^2+^ is sufficient to saturate the active binding sites with minimal competition. Under these conditions, the sorbent operates at near-maximum uptake per unit mass. However, as the adsorbent dose is progressively increased, a greater number of active sites are introduced into the system while the initial adsorbate concentration remains fixed. This surplus of sites leads to a phenomenon known as concentration gradient splitting. In this state, the fixed amount of available metal ions is distributed across an excessively large number of binding sites, the mass transfer of ions becomes less efficient, and the quantity of adsorbate sequestered per unit mass of the composite consistently declines 58. Therefore, the intermediate range of 0.05–0.1 g is identified as the optimal operational threshold, balancing maximum per-gram performance with the onset of mass-transfer limitations.

#### Influence of pH on adsorption capacity

**Fig. 10:**
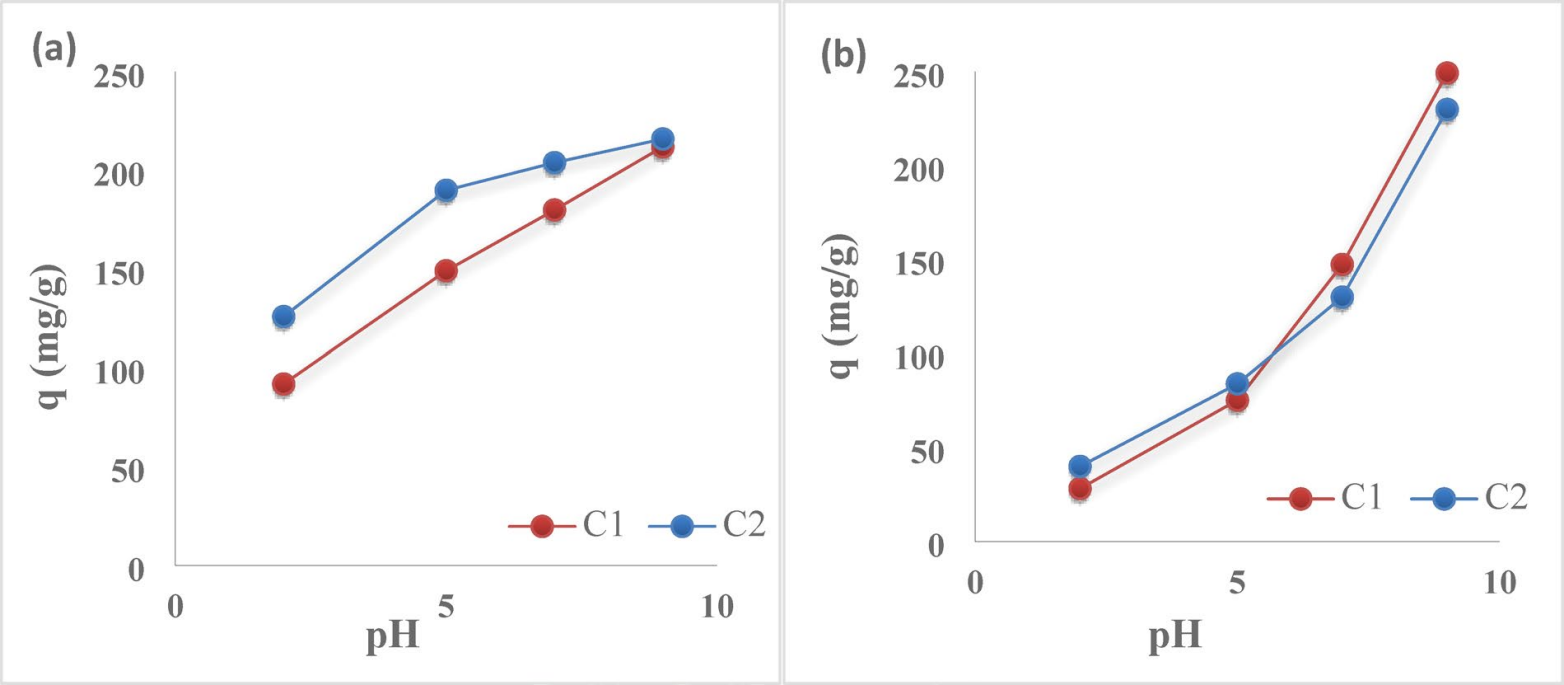
Variation in the adsorption capacity of Cu^2+^ (**a**) and Pb^2+^ (**b**) as a function of pH using 50 mg of synthesized composites C1 and C2. The batch trials were maintained at a fixed initial concentration of 150 mg/L in 100 mL of solution, with contact intervals of 30 min for Cu^2+^ and 60 min for Pb^2+^.

The adsorption capacities of composites C1 and C2 for Cu^2+^ and Pb^2+^ ions were heavily dependent on the initial solution pH. As illustrated in Fig. 10, a distinct enhancement in uptake capacity for both heavy metals was observed as the pH increased from 2 to 9. This performance trend is governed by a combination of competitive ion dynamics and surface charge modifications. At highly acidic conditions (pH < 3), the abundance of hydronium ions (H^+^) in the aqueous matrix competes aggressively with the divalent metal cations for available active sites. Conversely, as the pH rises toward near-neutral conditions, the decrease in H^+^ concentration mitigates competitive effects. Concurrently, the deprotonation of the composite’s surface functional groups imparts a net negative charge to the matrix, significantly boosting the electrostatic attraction and subsequent chemisorption of the positively charged Cu^2+^ and Pb^2+^ ions^54^.

#### Influence of initial metal concentration on adsorption capacity

**Fig. 11:**
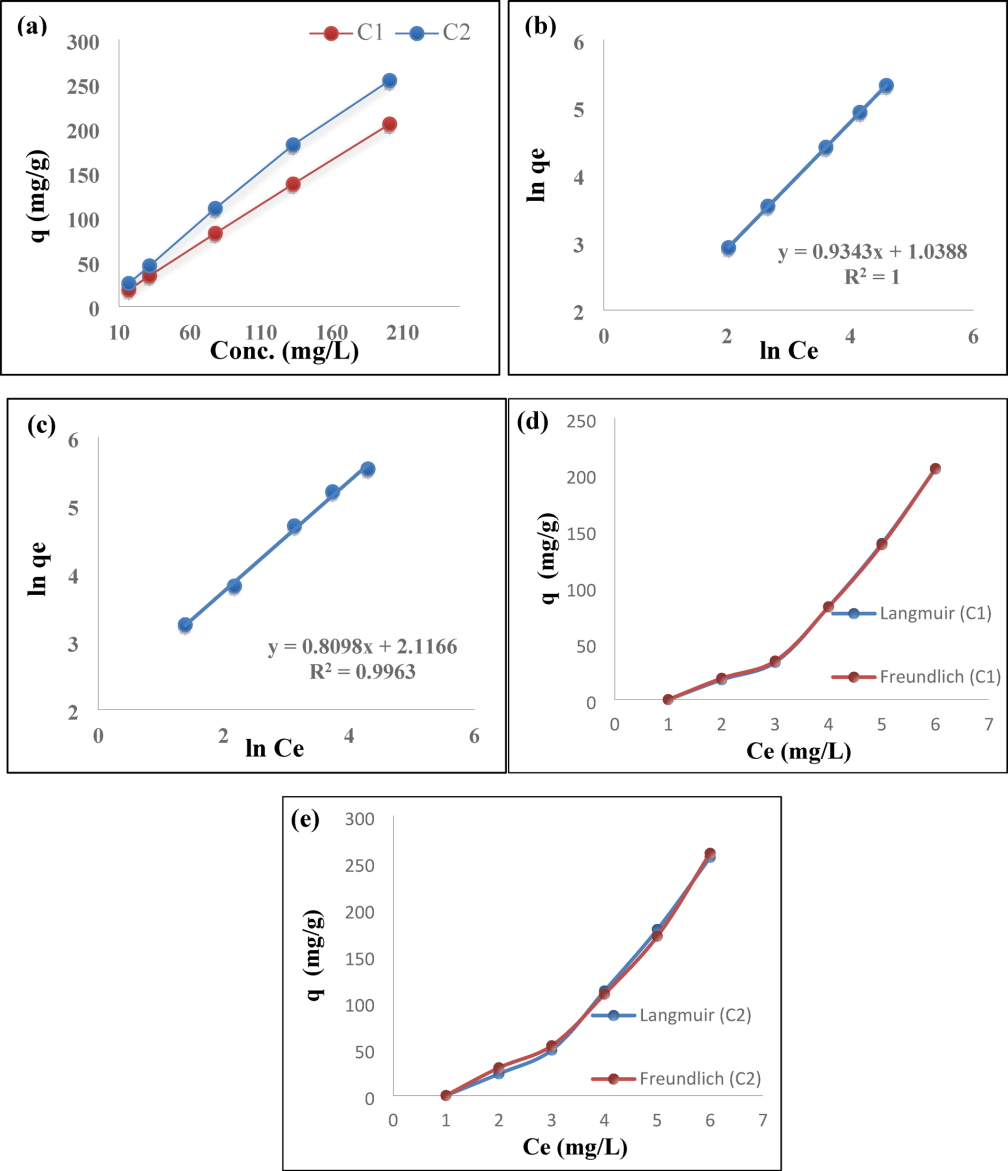
Adsorption equilibrium of Cu^2+^ onto bio-composites C1 and C2: (**a)** influence of initial ion concentration on uptake capacity, (**b**) linearized Freundlich isotherm plot for C1, (**c**) linearized Freundlich isotherm plot for C2, and corresponding non-linear Langmuir and Freundlich isotherm fits for (**d**) C1 and (**e**) C2. [Experimental conditions: adsorbent dosage = 50 mg/100 mL, contact time = 30 min].

**Fig. 12:**
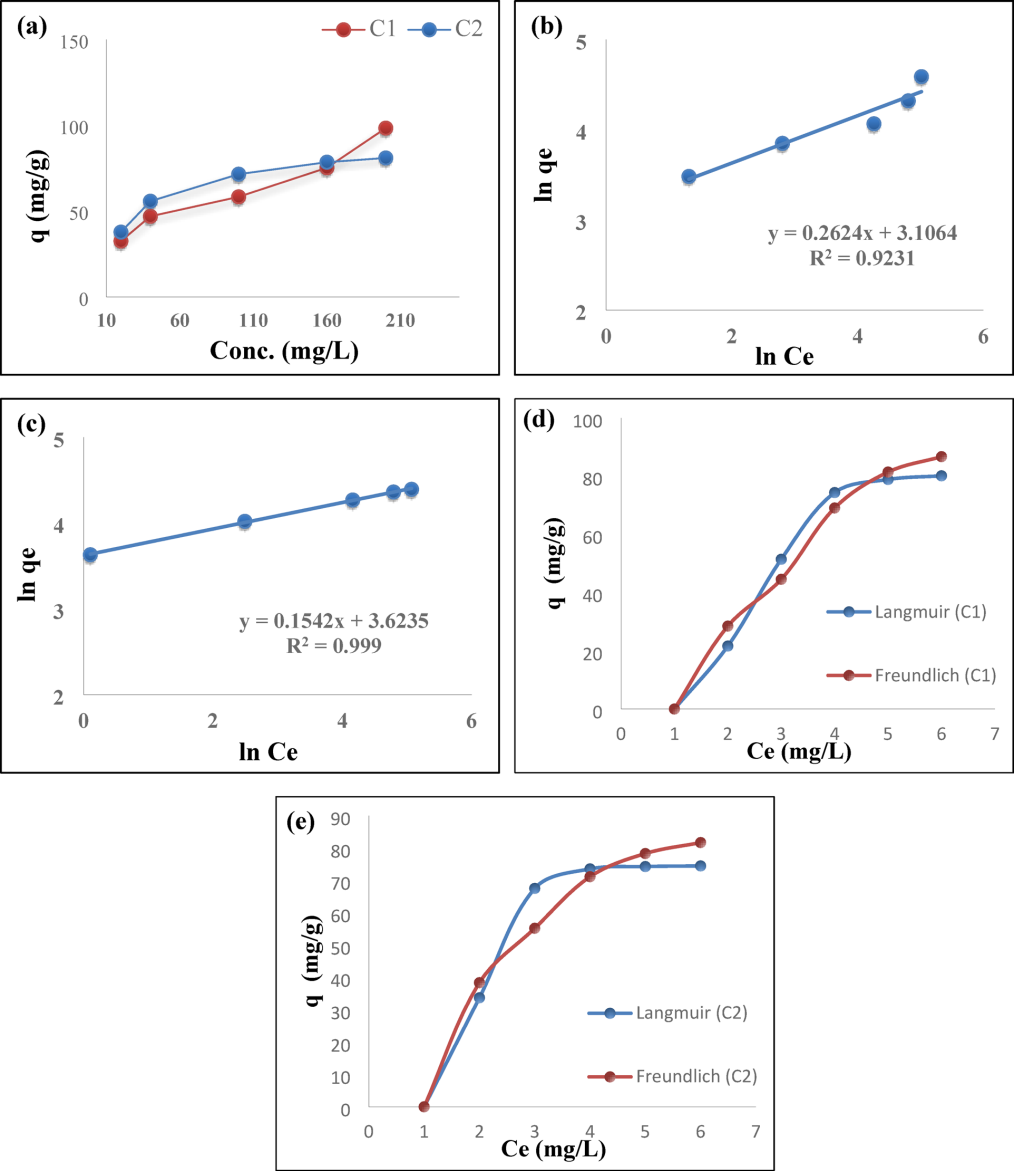
Adsorption equilibrium of Pb^2+^ onto bio-composites C1 and C2: (**a**) influence of initial ion concentration on uptake capacity, (**b**) linearized Freundlich isotherm plot for C1, (**c**) linearized Freundlich isotherm plot for C2, and corresponding non-linear Langmuir and Freundlich isotherm fits for (**d**) C1 and (**e**) C2. [Experimental conditions: adsorbent dosage = 50 mg/100 mL, contact time = 60 min].

The correlation between the initial solute concentration and the resultant uptake performance was evaluated to determine the maximum loading thresholds for the C1 and C2 composites, as illustrated in Figs. 11 and 12. These investigations utilized a constant sorbent mass of 50 mg while adjusting the initial concentrations of Cu^2+^ and Pb^2+^ within a range of 20 to 200 mg/L. The empirical data revealed a strong positive correlation between starting concentration and the adsorption capacity (q), demonstrating that these materials utilize their surface area more effectively as ion availability increases. Specifically, for copper ions, the capacity rose significantly from 19 to 205 mg/g for C1 and from 26 to 254 mg/g for C2 across the experimental range. A similar upward trend was noted for lead, where uptake values increased from 32 to 98 mg/g (C1) and from 38 to 81 mg/g (C2). This substantial elevation in q at higher concentrations is primarily credited to an intensified concentration gradient, which generates a more robust driving force for mass transfer. This gradient pressure facilitates the migration of metallic cations from the bulk aqueous phase to the active binding sites on the composite surface, effectively overcoming mass transfer resistance and activating sites that remain unoccupied at lower concentrations^54,59^.

To further define the interaction between the metal ions and the composite surfaces, the equilibrium datasets were analyzed using linear and nonlinear Langmuir, Freundlich, Temkin, and Dubinin–Radushkevich (D–R) models. In strict accordance with peer recommendations, relying solely on relative R^2^ values was avoided, and model suitability was rigorously cross-examined using absolute error functions namely, the Sum of Squared Errors (SSE), Root Mean Square Error (RMSE), and Chi-square ($\:{\chi\:}^{2}$). For the Cu^2+^ system, as shown in Table 3. the linearized plots initially suggested an exceptional fit with the Freundlich isotherm, with linear correlation coefficients (R^2^) reached 0.9999 for C1 and 0.9936 for C2, which superficially indicated a heterogeneous multilayer mechanism. However, the non-linear error analysis completely refined this mechanistic conclusion; the non-linear Langmuir model exhibited the absolute lowest error footprints (SSE = 4.2, $\:{\chi\:}^{2}$= 0.13 for C1; and SSE = 35.2, $\:{\chi\:}^{2}$= 0.60 for C2), outperforming the non-linear Freundlich model. This robustly confirms that Cu^2+^ sequestration occurs predominantly via uniform monolayer chemisorption onto a finite network of energetically equivalent active sites, yielding maximum non-linear adsorption capacities (q_max_) of 184.6 mg/g for C1 and 597.7 mg/g for C2 compared to 250 mg/g for C1 and 588 mg/g for C2 derived linearly. Conversely, as shown in Table 4 non-linear regression validated a distinct mechanistic pathway for the Pb^2+^ system. Although the linearized Langmuir and Freundlich plots displayed highly competitive coefficients of determination (R^2^ > 0.9 and R^2^ > 0.92, respectively), the non-linear Freundlich model emerged as the definitive mathematical match. This was particularly evident for the C2 matrix, which achieved an outstanding fit characterized by a non-linear R^2^ of 0.9989 and exceptionally low error indices (SSE = 1.3, RMSE = 0.53, and $\:{\chi\:}^{2}$ = 0.02). This rigorous, error-driven validation firmly establishes that Pb^2+^ immobilization is governed by multi-layer adsorption over the energetically heterogeneous surfaces of the hybrid bio-composites., with maximum Langmuir capacities recorded at 86 mg/g (C1) and 75.1 mg/g (C2) compared to 97 mg/g for C1 and 82 mg/g for C2 derived linearly^40,53,58^.

The favorability of the process is further confirmed by two diagnostic parameters: *n* > 1 in the Freundlich model and a separation factor R_L_ < 1 in the Langmuir model, both of which indicate highly favorable adsorption conditions^60^. Additionally, the affinity constant (b) results suggest that C2 possesses a significantly stronger affinity for both copper and lead ions compared to C1. To ensure a comprehensive equilibrium analysis and further explore the energetic distribution across the composite surfaces, both the linear and non-linear regression frameworks for the Temkin and Dubinin–Radushkevich (D–R) models were thoroughly scrutinized. However, the simultaneous analysis of both mathematical approaches reveals that neither framework provides an adequate representation of the experimental equilibrium data. The substantial conflict observed between the linear and non-linear parameters, along with the massive inflation of the absolute error functions, conclusively confirms that the adsorption system does not conform to the theoretical assumptions of these frameworks, thereby consolidating the definitive superiority of the primary best-fit isotherm models. Nevertheless, as detailed in Table 5, the maximum uptake capacities of the synthesized macro-composites significantly exceed those of numerous previously reported adsorbents, underscoring their superior performance in remediating contaminated water. Furthermore, while various biochar- and biomass-based adsorbents have been widely documented, they are typically utilized in loose powder forms, which severely restricts their practical scalability due to poor mechanical stability and complex post-treatment separation challenges. In contrast, this study introduces an innovative EVA-based macro-composite framework incorporating agricultural waste fillers, yielding a structurally robust and easily recoverable matrix. This distinctive architectural advantage, paired with the exceptional uptake capacities observed, highlights the strong practical viability and engineering scalability of these developed hybrid materials for advanced wastewater treatment.

**Table 3:** linear and nonlinear equilibrium isotherm constants for the adsorption of Cu^2+^ utilizing the synthesized composites C1 and C2.

Isotherms models constants											
Langmuir isotherm	q_max_ (mg/g)				b (L/mg)		*R* ^2^		SSE	RMSE	Chi-square
	Linear		Nonlinear		Linear	Nonlinear	Linear	Nonlinear	Nonlinear	Nonlinear	Nonlinear
C1	250		184.6		0.002	0.001	0.9312	0.9998	4.2	0.92	0.13
C2	588		597.7		0.01	0.01	0.9531	0.999	35.2	2.65	0.6

Freundlich isotherm	n				K_f_		R^2^		SSE	RMSE	Chi-square
	Linear		Nonlinear		Linear	Nonlinear	Linear	Nonlinear	Nonlinear	Nonlinear	Nonlinear
C1	1.1		1.07		2.8	2.9	1	0.9999	0.12	0.16	0.004
C2	1.2		1.3		8.3	10.6	0.9963	0.9936	229.8	6.78	2.5

Temkin isotherm	k_t_ (mol/L)				B		R^2^		SSE	RMSE	Chi-square
	Linear		Nonlinear		Linear	Nonlinear	Linear	Nonlinear	Nonlinear	Nonlinear	Nonlinear
C1	0.13		0.14		69.5	69.5	0.913	0.9084	2161	20.8	68.1
C2	0.25		0.25		78.3	78.35	0.9372	0.9372	2273.4	21.3	490.3

(D–R) isotherm	q_max_ (mg/g)		β		E (J/mol)		R^2^		SSE	RMSE	Chi-square
	Linear	Nonlinear	Linear	Nonlinear	Linear		Linear	Nonlinear	Nonlinear	Nonlinear	Nonlinear
C1	119.7	226.7	2 × 10^− 5^	0.0002	0.158		0.7793	0.9131	2050	20.25	NC
C2	147.7	264.8	6 × 10^− 6^	8.3 × 10^− 5^	0.288		0.7353	0.9119	3191	25.3	NC

NC,  not converged.

**Table 4:** linear and nonlinear equilibrium isotherm constants for the adsorption of Pb^2+^ utilizing the synthesized composites C1 and C2.

Isotherms models constants											
Langmuir isotherm	q_max_ (mg/g)				b (L/mg)		*R* ^2^		SSE	RMSE	Chi-square
	Linear		Nonlinear		Linear	Nonlinear	Linear	Nonlinear	Nonlinear	Nonlinear	Nonlinear
C1	97		86		0.047	0.09	0.9109	0.7152	742.8	12.2	13.7
C2	82		75.1		0.2	0.73	0.9977	0.8326	219	6.6	3.4

Freundlich isotherm	n				K_f_		R^2^		SSE	RMSE	Chi-square
	Linear		Nonlinear		Linear	Nonlinear	Linear	Nonlinear	Nonlinear	Nonlinear	Nonlinear
C1	3.8		3.3		22.34	19.2	0.9231	0.8805	311.8	7.9	4.4
C2	6.5		6.6		37.5	37.8	0.9990	0.9989	1.3	0.53	0.02

Temkin isotherm	k_t_ (mol/L)				B		R^2^		SSE	RMSE	Chi-square
	Linear		Nonlinear		Linear	Nonlinear	Linear	Nonlinear	Nonlinear	Nonlinear	Nonlinear
C1	1.75		1.75		14.8	14.8	0.8202	0.8202	468.9	9.68	6.6
C2	59.7		59.7		8.76	8.76	0.9952	0.9952	6.3	1.1	0.11

(D–R) isotherm	q_max_ (mg/g)		β		E (J/mol)		R^2^		SSE	RMSE	Chi-square
	Linear	Nonlinear	Linear	Nonlinear	Linear		Linear	Nonlinear	Nonlinear	Nonlinear	Nonlinear
C1	68.3	70.9	2 × 10^− 6^	2 × 10^− 6^	0.5		0.6207	0.4728	1375	16.58	19.7
C2	63.6	63.6	2 × 10^− 6^	2 × 10^− 6^	0.5		0.7757	0.5092	641.9	11.3	10.1

**Table 5:** Comparative Analysis of Cu^2+^ and Pb^2+^ Adsorption Capacities Across Various Sorbents.

Sorbents	Adsorption capacity (mg/g)		References
Cu^2+^			
SC-HA/C composite	9.2		^61^
activated carbon	17.09		^62^
CS–alginate beads	67.66		^63^
Modified chitosan polymeric compounds	88.49		^7^
Cross-linked chitosan grafted with polyaniline	131.58		^64^
Magnetic chitosan@bismuth tungstate coated by silver	181.8		^65^
C1	Linear	Nonlinear	Present work
	250	184.6	
C2	Linear	Nonlinear	Present work
	588	597.7	
Pb^2+^			
Callinectes sapidus biomass	31.44		^66^
Cellulose-MT-CBM bio sorbents	39.02		^67^
Cellulose	43.96		^68^
Polypyrrole-based activated carbon	50		^69^
*M. baccifera* activated charcoal	53.76		^70^
Sugar beet pulp	60		^71^
C1	Linear	Nonlinear	Present work
	97	86	
C2	Linear	Nonlinear	Present work
	82	75.1	

#### Synergistic mechanisms of Cu^2+^ and Pb^2+^ adsorption

The uptake of Cu^2+^ and Pb^2+^ by the synthesized C1 and C2 composites is characterized by a sophisticated interplay of concurrent chemical and physical phenomena. Kinetic modeling provides significant insight into this relationship; the rapid uptake rates and the superior fit of the pseudo-second-order equation indicate that the rate-limiting step is predominantly controlled by chemisorption. This process involves the formation of strong chemical bonds, likely through electron sharing or exchange between the metal ions and the active functional groups present on the composite surfaces. However, the equilibrium analysis introduces a more nuanced perspective on the energetic landscape of the system. The mean free energy (E) values calculated from the (D–R) isotherm is consistently low, falling well below the 8 kJ/mol threshold typically associated with pure chemical bonding. These values suggest a substantial physisorption component, where electrostatic attractions and ion-exchange mechanisms facilitate the initial migration and stabilization of the heavy metals within the material’s porous architecture. This dual-mechanism profile is further validated by the system’s response to varying experimental parameters. At elevated initial concentrations, a robust concentration gradient acts as a powerful driving force, enabling the ions to overcome the mass transfer resistance inherent in physical diffusion. Conversely, as the system reaches equilibrium, the eventual saturation of the surface confirms a finite number of chemical binding sites, underscoring the chemisorptive limit. By leveraging this hybrid mechanism, the stability of chemical complexation combined with the rapid mass transfer of physical attraction^50,54,72^.

## Conclusion

This study investigates the development of sustainable ethylene-vinyl acetate (EVA) hybrid bio-composites designed for the efficient removal of Cu^2+^ and Pb^2+^ ions from aqueous environments. Two specific bio-composites, C1 (EVA/biochar) and C2 (EVA/sugar beet pulp waste), were fabricated using a melt-blending technique that incorporates agricultural residues as functional fillers. Extensive characterization through SEM–EDX, FTIR, TGA, and DSC confirmed successful filler integration and strong interfacial compatibility within the EVA matrix, ensuring structural integrity and enhanced surface properties. Adsorption performance was evaluated by analyzing the influence of contact time, adsorbent dosage, and initial metal concentrations. Both materials demonstrated rapid removal kinetics, reaching equilibrium within 30 min for Cu^2+^ and 60 min for Pb^2+^. The biochar-based composite (C1) exhibited exceptional performance, yielding maximum experimental adsorption capacities for Cu^2+^ and Pb^2+^ of 250 mg/g and 97 mg/g via linear fitting, and 184.6 mg/g and 86 mg/g via non-linear fitting, respectively. In comparison, the sugar beet pulp composite (C2) recorded linear adsorption capacities of 588 mg/g for Cu^2+^ and 82 mg/g for Pb^2+^, while reaching non-linear capacities of 597.7 mg/g and 75.1 mg/g for the respective heavy metal. The systematic application of both linear and non-linear regression frameworks provided a rigorous mathematical validation of the process, offering deeper insights into the underlying chemisorption dynamics and multi-step diffusion mechanisms. Ultimately, the synergistic effect of the EVA matrix and bio-based fillers produced a stable, high-capacity material, positioning these agricultural waste-derived composites as promising candidates for sustainable heavy metal remediation in water treatment.

## Supplementary Information

Below is the link to the electronic supplementary material.

Supplementary Material 1

## Data Availability

The data used during the current study are available from the corresponding author upon reasonable request.
