# Supplementary material for: Sustainable EVA-based hybrid bio-composites derived from agricultural waste for efficient heavy metal removal
Source: Sci Rep. 2026 Jul 20;16:22671. doi: 10.1038/s41598-026-62480-9 (PMC13385710; doi:10.1038/s41598-026-62480-9)
Supplement: Supplementary file 1 — Supplementary Material 1 [file 41598_2026_62480_MOESM1_ESM.docx]

**Supplementary Information (S1)**

**Sustainable EVA-Based Hybrid Bio-Composites Derived from Agricultural Waste for Efficient Heavy Metal Removal**

**Kholod H. Kamal ^1^, Mahmoud E. Abd El-Aziz^2,^** ***, Ahmed Haroun^3^**

^1^ Water Pollution Research Department, National Research Centre, 33 El Bohouth Str, Dokki, Giza, Egypt, P.O. 12622

^2^ Polymers and Pigments Department, National Research Centre, 33 El Bohouth Str, Dokki, Giza, Egypt, P.O. 12622.

**^3^** Chemical Industreies Research Institute, National Research Centre, 33 El Bohouth Str, Dokki, Giza, Egypt, P.O. 12622.

*Corresponding author: Mahmoud E. Abd El-Aziz, email: [mahmoud_essam12@yahoo.com](mailto:mahmoud_essam12@yahoo.com)

**S1:** Potential kinetic and isotherm models for Cu²⁺ and Pb²⁺ eradication

| **Kinetics Models** | | | |
| --- | --- | --- | --- |
| **Models** | **Equations** | **Variables** | **Remarks** |
| Pseudo-first-order | ***Linear:***  *Log* (*q_e_*- *q_t_*) = *log* *q_e_* – (*k_1_*/2.303) *t*  ***Nonlinear****:*  $q_{t}=q_{e}(1-e^{-k_{1}t})$  *Here;*  *q_t_ = (C_0_ – C_t_) * (V/M)* | *q_e_*: equilibrium sorption capacity (mg/g)  *q_t:_* capacity of sorption (mg/g) at a time (*t*, min)  *k_1_* _:_ the rate constant (min^-1^)  *V*: volume of solution (L)  *M*: mass of the sorbent added (g) | ----- |
| Pseudo-second-order | ***Linear:***  *t*/*q_t_* = 1/*k_2_q_e_* + (1/*q_e_*) *t*  ***Nonlinear****:*  $q_{t}=\frac{k_{2} q_{e}^{2} t}{1+ k_{2}q_{e} t}$ | *k_2_*_:_ the rate constant (g/mg. min) | ------ |
| Intra-particle diffusion model | ***Linear:***  *q_t_* = *k_p_* (*t*)^0.5^ + *c*  ***Nonlinear****:*  $q_{t}=k_{p}t^{0.5}+C$ | *k_p_*: intra-particle diffusion rate (mg. g^-1^min^1/2^)  *C*: constant | ------ |
| Elovich | ***Linear:***  *q_t_* = (1/*β*) *ln* (*αβ*) + (1/*β*) *ln* (*t*)  ***Nonlinear****:*  $q_{t}=\frac{1}{\beta} ln(1+\alpha\beta t)$ | *β:* the desorption constant (mg. g^-1^.min)  *α*: the initial adsorption rate (mg. g^-1^.min^-1^) | ------ |
| **Isotherms Models** | | | |
| Langmuir isotherm | ***Linear:***  *C_e_*/*q_e_* = 1/*bq_max_* + (1/*q_max_*) *C_e_*  *R_L_* = 1/(1+*bC_0_*)  ***Nonlinear****:*  $q_{e}=\frac{q_{max}bC_{e}}{1+bC_{e}}$ | *b:* the Langmuir constant (l/mg)  *q_max_*: the maximum sorption capacity (mg/g)  *R_L_* : separation factor | *R_L_* = 1: shows linear adsorption  *R_L_* = 0: illustrates irreversible  *R_L_*> 1: represents unfavorable adsorption  0<*R_L_*< 1 : indicated good adsorption |
| Freundlich isotherm | ***Linear:***  *ln* *q_e_* = *ln* *k_f_* + (1/*n*) *ln* *C_e_*  ***Nonlinear****:*  $q_{e}=K_{f}C_{e}^{1/n}$ | *k_f_:* the Freundlich constant  *n*: the strength of adsorption | *n*=1: characterizes linear adsorption  *n*<1: represents the chemical process  *n*>1: designates the physical process |
| Temkin isotherm | ***Linear:***  *q_e_* = (*RT/b*) *ln* *k_t_* + (*RT/b*) *ln* *C_e_*  ***Nonlinear****:*  $q_{e}=\frac{RT}{b}ln(K_{t}C_{e})$ | *b****:*** the Temkin constant corresponding to the adsorption heat  *R***:** the universal gas constant (0.00813 kJ/mol K)  *K_t_:* the equilibrium binding constant (mol/l)  *T*: the temperature (K) | ------- |
| (D–R) isotherm | ***Linear:***  *ln* *q_e_* = *ln* *q_m_* - *βɛ^2^*  *ɛ* = *RT* *ln* (1 + 1/*C_e_*)  *E* = 1/(2*β*)^0.5^  ***Nonlinear****:*  $q_{e}=q_{m}e^{-\betaɛ^{2}}$ | *β*: the activity coefficient  *Ɛ*: the Polanyi potential  *E* : Sorption energy | ------- |
